# An iron detection system determines bacterial swarming initiation and biofilm formation

**DOI:** 10.1038/srep36747

**Published:** 2016-11-15

**Authors:** Chuan-Sheng Lin, Yu-Huan Tsai, Chih-Jung Chang, Shun-Fu Tseng, Tsung-Ru Wu, Chia-Chen Lu, Ting-Shu Wu, Jang-Jih Lu, Jim-Tong Horng, Jan Martel, David M. Ojcius, Hsin-Chih Lai, John D. Young

**Affiliations:** 1 Department of Medical Biotechnology and Laboratory Science, Chang Gung University, Taoyuan, Taiwan, Republic of China; 2 Department of Biochemistry and Molecular Biology, Chang Gung University, Taoyuan, Taiwan, Republic of China; 3 Research Center of Bacterial Pathogenesis, Chang Gung University, Taoyuan, Taiwan, Republic of China; 4 Center for Molecular and Clinical Immunology, Chang Gung University, Taoyuan, Taiwan, Republic of China; 5 Department of Respiratory Therapy, Fu Jen University, New Taipei City, Taiwan, Republic of China; 6 Department of Internal Medicine, Chang Gung Memorial Hospital, Taoyuan, Taiwan, Republic of China; 7 Department of Laboratory Medicine, Chang Gung Memorial Hospital, Taoyuan, Taiwan, Republic of China; 8 Department of Biomedical Sciences, University of the Pacific, Arthur Dugoni School of Dentistry, San Francisco, United States of America; 9 Research Center for Industry of Human Ecology, Chang Gung University of Science and Technology, Taoyuan, Taiwan, Republic of China; 10 Graduate Institute of Health Industry Technology, Chang Gung University of Science and Technology, Taoyuan, Taiwan, Republic of China; 11 Laboratory of Cellular Physiology and Immunology, Rockefeller University, New York, United States of America; 12 Biochemical Engineering Research Center, Ming Chi University of Technology, New Taipei City, Taiwan, Republic of China

## Abstract

Iron availability affects swarming and biofilm formation in various bacterial species. However, how bacteria sense iron and coordinate swarming and biofilm formation remains unclear. Using *Serratia marcescens* as a model organism, we identify here a stage-specific iron-regulatory machinery comprising a two-component system (TCS) and the TCS-regulated iron chelator 2-isocyano-6,7-dihydroxycoumarin (ICDH-Coumarin) that directly senses and modulates environmental ferric iron (Fe^3+^) availability to determine swarming initiation and biofilm formation. We demonstrate that the two-component system RssA-RssB (RssAB) directly senses environmental ferric iron (Fe^3+^) and transcriptionally modulates biosynthesis of flagella and the iron chelator ICDH-Coumarin whose production requires the *pvc* cluster. Addition of Fe^3+^, or loss of ICDH-Coumarin due to *pvc* deletion results in prolonged RssAB signaling activation, leading to delayed swarming initiation and increased biofilm formation. We further show that ICDH-Coumarin is able to chelate Fe^3+^ to switch off RssAB signaling, triggering swarming initiation and biofilm reduction. Our findings reveal a novel cellular system that senses iron levels to regulate bacterial surface lifestyle.

Iron is essential for many cellular processes^1^. While low iron bioavailability is a limiting factor for cell survival in hostile environments, excess iron within the cell is toxic due in part to the formation of hydroxyl radicals through Fenton reactions^2^. Iron also serves as a stress signal that regulates microbial physiology, such as susceptibility to antibiotics^3^. Competition between the host and pathogens for limited iron resources may determine infections outcome^4^. Many homeostatic systems thus tightly control intracellular iron concentration in bacteria in order to allow adaptation to ever-changing environments^5^,^6^,^7^.

Swarming and biofilm formation are two typical multicellular behaviors of bacteria living on a surface^8^. Bacteria within biofilms embedded in an extracellular matrix undergo cellular differentiation and may acquire resistance to environmental stress and host immune responses^9^,^10^. On the other hand, swarming, which is observed in various bacterial species, represents a rapid, cell density-dependent, flagellum-driven movement of bacteria on a surface, and is closely associated with antibiotic resistance and production of virulence factors^11^,^12^,^13^,^14^,^15^. Swarming is characterized by a non-motile lag phase and an active migration phase associated with metabolic and morphological changes^11^,^16^,^17^. Several studies have identified regulatory systems that accelerate swarming migration velocity by increasing flagella and biosurfactant production^18^,^19^,^20^,^21^. However, the cellular mechanism underlying initiation of swarming and biofilm formation remains incompletely understood.

Swarming initiation is associated with changes in the expression of genes involved in the metabolism, acquisition and transport of iron in various bacterial species^14^,^22^,^23^. Iron limitation induces cell differentiation in swarming^24^, and disruption of the iron acquisition system affects swarming^25^. Additionally, iron chelation reduces biofilm formation, whereas iron overloading promotes biofilm formation^26^,^27^,^28^. Further identification of the sensor in response to environmental iron and downstream signaling may help us to understand the transition between swarming and biofilm formation.

Two-component systems (TCSs), typically composed of histidine sensor kinases and cognate response regulators, are among the most sophisticated signaling systems used by bacteria to sense and react to environmental stimuli. Control of phosphotransfer from membrane-bound histidine sensor kinases to response regulator in TCSs offers bacteria the ability to adapt to a wide range of environmental conditions^29^,^30^,^31^. We previously identified a TCS, called RssA-RssB (RssAB), whose activation is involved in coordinating the development of surface multicellularity as well as virulence in *Serratia marcescens*^32^,^33^,^34^,^35^,^36^. Here we show that RssA directly senses ferric iron (Fe^3+^) via its periplasmic region, and that this interaction leads to RssB phosphorylation. The Fe^3+^ chelator 2-isocyano-6,7-dihydroxycoumarin (ICDH-Coumarin), whose biosynthesis is under transcriptional control of RssAB signaling, is shown to fine-tune RssAB-modulated swarming initiation and biofilm formation by controlling extracellular Fe^3+^ availability. Our results show that extracellular iron sensing by a TCS regulates multicellular behaviors in bacteria.

## Results

### Fe^3+^ regulates swarming initiation and biofilm formation

**Figure 1:**
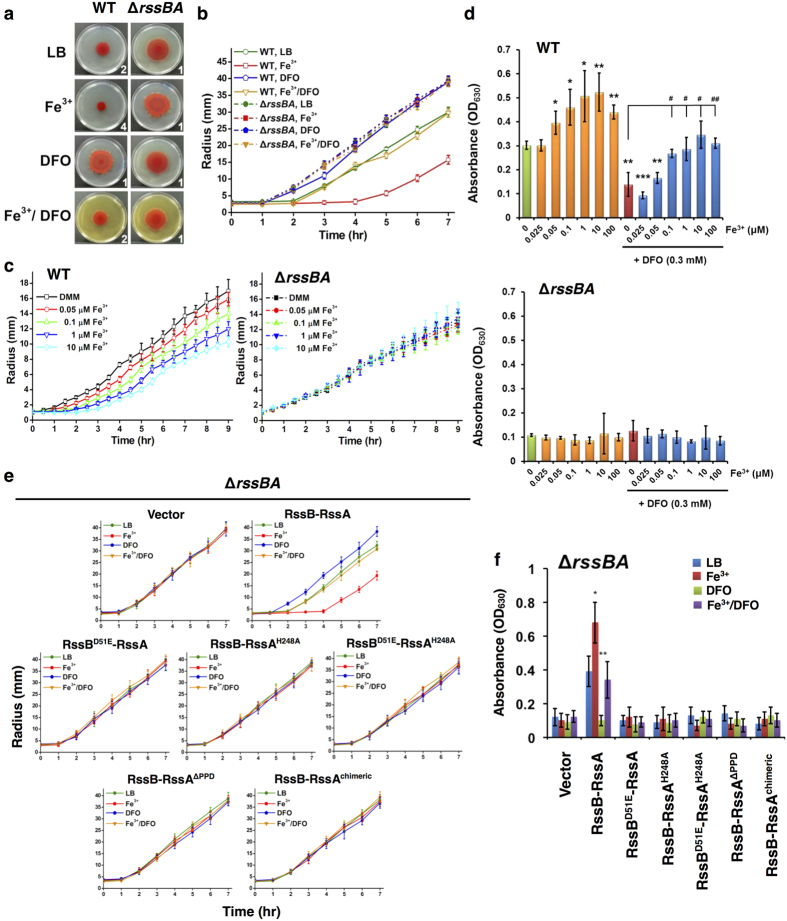
Fe^3+^ controls swarming initiation and biofilm formation in *S. marcescens* through the TCS RssAB. **(a**,**b)** Swarming pattern **(a)** and migration radius **(b)** of WT and Δ*rssBA S. marcescens* on LB swarming plates containing Fe^3+^ (100 μM) and/or DFO (0.3 mM). **(c)** Swarming migration radius of WT and Δ*rssBA* on DMM swarming plates containing Fe^3+^ at the indicated concentration (0–10 μM). The number in the lower right corner of each swarming plate image represents the duration of the lag phase in hours. Migration radius (mm) corresponds to mean ± SEM (n = 3). ND, not detected. **(d)** As mentioned in Methods, biofilm of WT and Δ*rssBA S. marcescens* in LB broth containing the indicated concentration of Fe^3+^ and/or DFO (0.3 mM) was quantified by monitoring absorbance at 630 nm. **(e**,**f)** In LB condition with or without Fe^3+^ (100 μM) and DFO (0.3 mM), swarming radius **(e)** and biofilm quantification **(f)** of Δ*rssBA* harboring the vector pACYC184 or the recombinant pACYC184 plasmid containing different constructs of RssB and RssA driven by their native promoters. RssB^D51E^ and RssA^H248A^: constructs with point mutations at conserved phosphorylation sites; RssA^ΔPPD^: RssA with deletion in periplamic domain (PPD, amino acids 32–163; RssA^chimeric^: a chimeric RssA whose periplasmic domain was replaced with the periplasmic domain of QseC. The results represent means ± SEM from three independent experiments (n = 3). Statistical analysis was performed using one-way ANOVA. For Fig. 1d, *, ** and *** represent P < 0.05, 0.01, 0.001, and 0.0001, respectively, compared to the untreated sample. ^#^ and ^##^ represent P < 0.05 and 0.01, respectively, compared to the group treated with DFO but without Fe^3+^. For Fig. 1f, *P < 0.05; **P < 0.01 compared to LB group.

To examine whether iron regulates multicellular behavior, we used the wild-type (WT) *S. marcescens* CH-1 strain, which exhibits canonical swarming consisting of a non-motile lag phase and a motile migration phase^35^ on Luria-Bertani (LB) swarming plates. We observed that ferric iron (Fe^3+^) availability determines the timing of swarming initiation in the WT strain (Fig. 1a,b), without affecting swarming expansion rate (Supplementary Fig. 1). Fe^3+^ depletion by the Fe^3+^ chelator deferoxamine mesylate (DFO) reduced lag phase duration and induced early swarming initiation (Fig. 1a,b). On the other hand, Fe^3+^ supplementation (100 μM) prolonged the lag phase and delayed swarming initiation, which was restored by co-administration of Fe^3+^ and DFO (Fig. 1a,b). Of note, when growing on iron-limiting, defined minimal medium (DMM) swarming plates, no swarming lag phase was observed, while addition of Fe^3+^ dose-dependently extended the lag phase to 2 hrs in WT bacteria (Fig. 1c). These results suggest that Fe^3+^ may control swarming initiation.

Addition of divalent cations such as Mg^2+^, Ca^2+^, Zn^2+^ and Co^2+^ produced no significant impact on swarming in *S. marcescens* (Supplementary Fig. 2a). Similarly to the effects of Fe^3+^, Fe^2+^ inhibited swarming and restored swarming induced by the metal-ion-chelator EDTA (Supplementary Fig. 2a). These effects were abrogated by the Fe^2+^ chelator 2,2′-dipyridyl (2,2′-DP) and the Fe^3+^ chelator DFO, while the effects of Fe^3+^ were abolished only by DFO (Supplementary Fig. 2b). Furthermore, Fe^3+^ and Fe^2+^-mediated repression of swarming was eliminated by addition of the reducing reagent sodium ascorbate^37^ (Supplementary Fig. 2c; ASC), suggesting that Fe^3+^ rather than Fe^2+^ is the main factor that delays swarming initiation.

The inverse relationship between swarming motility and biofilm formation^8^ led us to examine the effects of iron on biofilm formation. As expected, Fe^3+^ supplementation increased biofilm formation in a dose-dependent manner, and the effect of Fe^3+^ supplementation was inhibited by the addition of the Fe^3+^ chelator DFO (Fig. 1d). We concluded that extracellular Fe^3+^ concentration controls swarming initiation and biofilm formation in *S. marcescens*.

### The TCS RssAB is required for Fe^3+^-mediated regulation of swarming initiation and biofilm formation in *S. marcescens*

We previously reported that the TCS RssAB is temporally activated during the swarming lag phase and delays swarming initiation in *S. marcescens*^35^. We thus investigated whether RssAB mediates the effect of Fe^3+^ on swarming initiation. Deletion of the *rssBA* locus in WT *S. marcescens* abolished the effects of both iron and iron chelators on swarming initiation, and the *rssBA* deletion mutant (Δ*rssBA*) constitutively displayed early swarming initiation on LB swarming plates, 1 hr earlier than the WT strain (Fig. 1a,b and Supplementary Fig. 2; Δ*rssBA*). In addition, the ability of Fe^3+^ to prolong the swarming lag phase on iron-limiting DMM swarming plates and to induce biofilm formation were not detected in the Δ*rssAB* strain (Fig. 1c,d; Δ*rssBA*). Episomal expression of a wild-type RssB-RssA construct in Δ*rssBA* bacteria complemented the iron responsiveness for both swarming (Fig. 1e and Supplementary Fig. 3) and biofilm formation (Fig. 1f). However, expression of RssB-RssA constructs harboring mutations at conserved phosphorylation sites, either at aspartate 51 (D51) for RssB or histidine 248 (H248) for RssA^36^, failed to rescue iron irresponsiveness in Δ*rssBA* bacteria, when either swarming (Fig. 1e and Supplementary Fig. 3) or biofilm formation was analyzed (Fig. 1f). These results demonstrate that RssAB signaling is responsible for the effects of Fe^3+^ on swarming initiation and biofilm formation in *S. marcescens*.

### Fe^3+^ activates RssAB signaling during swarming and biofilm formation

**Figure 2:**
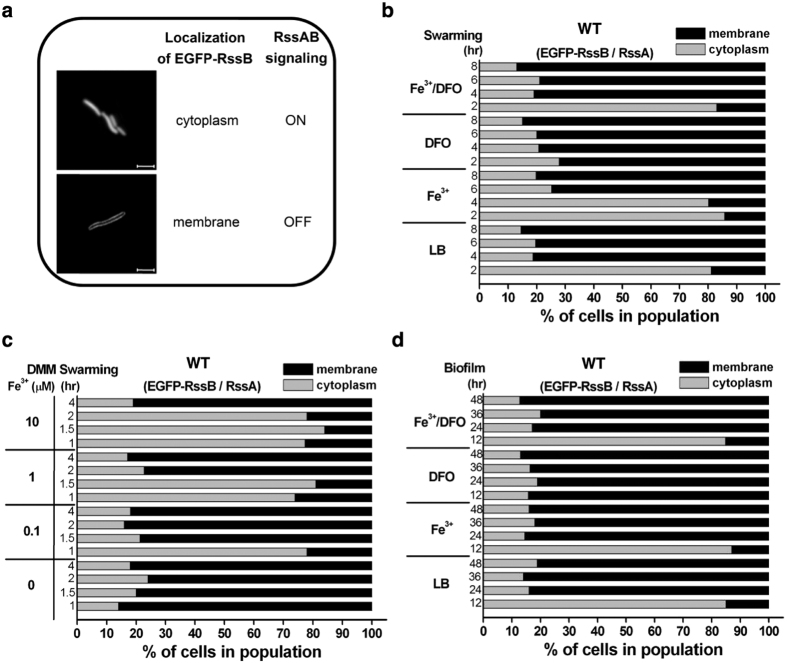
Fe^3+^ availability regulates RssAB signaling status during swarming and biofilm development. **(a)** Representative image of cytolocalization of EGFP-tagged RssB. Cytoplasmic and membrane location of EGFP-RssB indicates ON (activated) and OFF (inactivated) RssAB signaling status, respectively. Scale bar, 3 μm. **(b)** WT *S. marcescens* harboring the pEGFP-RssBA::Sm plasmid encoding EGFP-RssB and RssA was used to evaluate the state of RssAB signaling during swarming (2–8 hr). Swarming assays were performed on LB plates containing 0.1% arabinose with or without Fe^3+^ (100 μM) and DFO (0.3 mM). Cellular localization of EGFP-RssB was monitored to quantify the percentage of activated and inactivated RssAB signaling. **(c)** Quantification of RssAB signaling status during swarming progression (1–4 hr) on DMM swarming plates supplemented with Fe^3+^ at the indicated concentration (0–10 μM). **(d)** Quantification of RssAB signaling status during biofilm development (12–48 hr) in LB condition with or without Fe^3+^ (100 μM) and DFO (0.3 mM). Percentage of cell type is shown as mean from three independent experiments performed in triplicate. At least 200 cells were counted for each assay condition.

To investigate whether environmental Fe^3+^ modulates RssAB, we monitored RssAB signaling in response to Fe^3+^ availability by examining the cytolocalization of enhanced green fluorescent protein (EGFP)-tagged RssB during swarming and biofilm formation (Fig. 2a)^35^. On LB swarming plates, dispersal of EGFP-RssB in the cytosol, which indicates activation of RssAB signaling (ON), was observed 2 hr in the swarming lag phase (Fig. 2b; LB-2 hr). During the surface migration phase of swarming, EGFP-RssB was detected at the cell membrane, which represents the resting state (OFF) of RssAB (Fig. 2b; LB-4, 6, 8 hr). While Fe^3+^ supplementation extended RssAB signaling activation to 4 hr (Fig. 2b; Fe^3+^), DFO-mediated Fe^3+^ depletion resulted in constitutive OFF signaling during the entire swarming period (Fig. 2b; DFO). Addition of both Fe^3+^ and DFO in LB swarming plates did not affect RssAB signaling state, indicating that the lag period was specifically extended by Fe^3+^ on LB swarming plates (Fig. 2b; Fe^3+^/DFO). In contrast to LB swarming plates, RssAB signaling was constitutively OFF on iron-limiting DMM swarming plates (Fig. 2c), where no lag phase was observed (Fig. 1c). Iron supplementation induced RssAB signaling and dose-dependently extended the duration of RssAB activation up to 2 hrs (Fig. 2c), which also prolonged the duration of the lag phase in swarming development (Fig. 1c).

We previously showed^35^ that RssAB signaling is specifically activated during the early stage of biofilm formation (Fig. 2d; LB-12 hr) and is deactivated in mature biofilms (Fig. 2d; LB-24, 36 and 48 hr). Here, we found that, while addition of Fe^3+^ did not change the timing of RssAB activation (Fig. 2d; Fe^3+^), Fe^3+^ depletion by DFO abrogated activation of RssAB signaling (Fig. 2d; DFO), and this effect could be restored by Fe^3+^ supplementation (Fig. 2d; Fe^3+^/DFO). Together with the observation that RssAB signaling is required for Fe^3+^-mediated modulatory effects on swarming and biofilm formation (Fig. 1e,f and Supplementary Fig. 3), we conclude that Fe^3+^ controls RssAB signaling to regulate swarming and biofilm formation.

### RssAB directly senses Fe^3+^ at the nanomolar level

To examine how Fe^3+^ affects RssAB signaling, we first studied RssAB signaling in response to Fe^3+^ addition in iron-limiting DMM broth, which allowed us to assess the status of RssAB signaling in real time. While RssAB signaling was constitutively OFF in DMM broth (Supplementary Fig. 4a), addition of Fe^3+^ immediately activated RssAB signaling for at least 60 min, and this effect was reverted by DFO (Supplementary Fig. 4b). Of note, replacement of Fe^3+^-treated bacterial culture broth with mock-treated culture broth deactivated RssAB signaling (Supplementary Fig. 4c), indicating that extracellular Fe^3+^ alters the state of RssAB signaling. We further demonstrated that Fe^3+^ at a concentration of 50 nM was sufficient to activate RssAB signaling (Supplementary Fig. 4d), consistent with the observation that 50 nM Fe^3+^ could prolong the duration of the lag phase on DMM swarming plates (Fig. 1c; WT).

**Figure 3:**
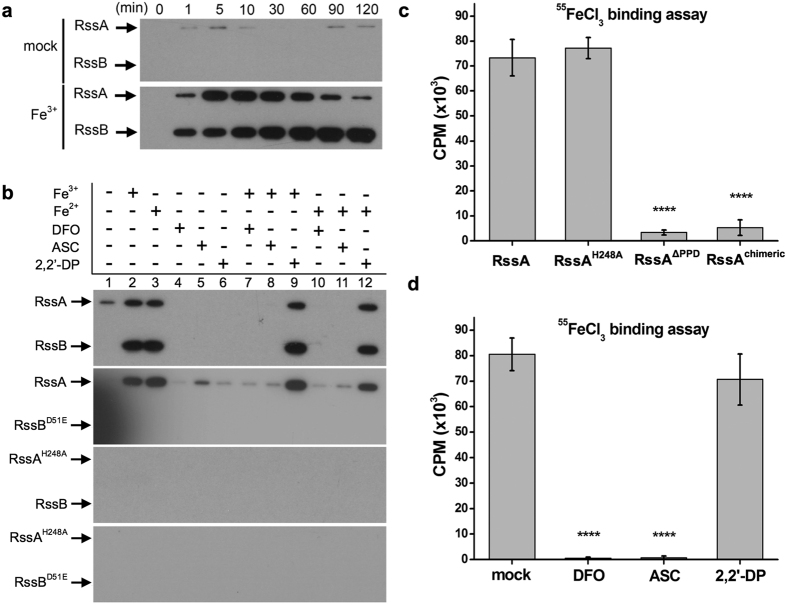
RssA binds Fe^3+^ through its periplasmic domain and transphosphorylates RssB. **(a)** His-tagged RssA and RssB reconstituted in liposomes with or without Fe^3+^ was supplemented with [γ^32^P]ATP (50 μCi), collected at the indicated time points, and examined by radiography imaging. **(b)** Liposomes containing His-tagged RssA, RssB, nonphosphorylatable RssA or RssB were harvested 30 min after addition of [γ^32^P]ATP and examined by radiography imaging. **(c)** His-tagged RssA, RssA^H248A^ (His248 mutated to Ala), RssA^ΔPPD^ (RssA with deletion in periplasmic domain), and RssA^chimeric^ (a chimeric RssA whose periplasmic domain was replaced with the periplasmic domain of QseC) were reconstituted in liposomes containing 500 nM ^55^FeCl_3_. After incubation, disrupted liposomes passed through NTA column to remove unbound ^55^FeCl_3_. Iron-bound membrane proteins were eluted and subjected to radioactivity analysis of liquid scintillation counting (counts per min, CPM). **(d)** His-tagged periplasmic domain of RssA was incubated with ^55^FeCl_3_ and mock (distilled water), DFO (0.3 mM), ASC (0.3 mM), or 2,2′-DP (0.3 mM). Radioactivity was determined in the periplasmic domain eluted from Ni^2+^-NTA column. Statistical analysis was performed using one-way ANOVA. For Fig. 1c, ****P-value < 0.0001, compared to RssA group. For Fig. 1d, *** and **** correspond to *P*-values < 0.001 and <0.0001, respectively, compared to mock group.

To address whether Fe^3+^ triggers RssAB transphosphorylation, we performed liposome-based radiography phosphorylation assays by reconstituting purified His-tagged RssA into liposomes under various iron conditions. We found that as soon as 1 min after exposure to [γ^32^P]ATP, autophosphorylation of RssA occurred in the presence of Fe^3+^, followed by phosphotransfer to RssB (Fig. 3a). Fe^3+^-induced RssAB transphorylation was inhibited by co-treatment with the Fe^3+^ chelator DFO (Fig. 3b). While the presence of Fe^2+^ triggered RssA autophosphorylation, co-treatment with the reducing reagent ASC or the Fe^3+^ chelator DFO (but not the Fe^2+^ chelator 2,2′-DP) prevented RssA autophosphorylation (Fig. 3b). Importantly, Fe^3+^-induced phosphorelay was largely dependent on the conserved phosphorylation sites of RssA and RssB (Fig. 3b), consistent with our observation that only the expression of functional RssAB could restore the effects of Fe^3+^ on swarming migration (Fig. 1e and Supplementary Fig. 3), biofilm formation (Fig. 1f), and signaling activation (Supplementary Fig. 5) in Δ*rssBA* bacteria.

As the periplasmic region of sensor kinases is generally responsible for sensing environmental cues^31^, we prepared a plasmid construct harboring RssA without the periplasmic domain (RssA^ΔPPD^) to test its function. Expression of RssA without the periplasmic domain (RssA^ΔPPD^) failed to rescue the phenotypes of Δ*rssBA* to Fe^3+^ in swarming initiation (Fig. 1e and Supplementary Fig. 3), biofilm formation (Fig. 1f), and RssAB signaling (Supplementary Fig. 5). Using the ^55^FeCl_3_ binding assay, we observed that full-length RssA could bind to Fe^3+^, whereas RssA^ΔPPD^ could not (Fig. 3c,d). Additionally, free Fe^3+^, in the absence of DFO or ASC, could directly bind to the purified periplasmic domain of RssA, indicating that the periplamic domain of RssA is responsible for Fe^3+^ binding (Fig. 3d). To test the specificity of the RssA periplasmic domain to Fe^3+^, we constructed a chimeric RssA (RssA^chimeric^) in which the periplasmic region of RssA was replaced by the corresponding region of QseC, a sensor kinase not involved in iron sensing^38^. RssA^chimeric^ did not interact with Fe^3+^ (Fig. 3c) and failed to restore the swarming lag period (Fig. 1e, Supplementary Fig. 3), biofilm formation (Fig. 1f), or Fe^3+^ responsiveness (Supplementary Fig. 5) in Δ*rssBA* bacteria. Collectively, these results indicate that Fe^3+^ directly and specifically binds to the periplasmic region of RssA, thereafter triggering RssAB signaling and regulating swarming initiation and biofilm formation.

### Identification of the RssAB-regulated *pvc* cluster and its involvement in swarming and biofilm formation

**Figure 4:**
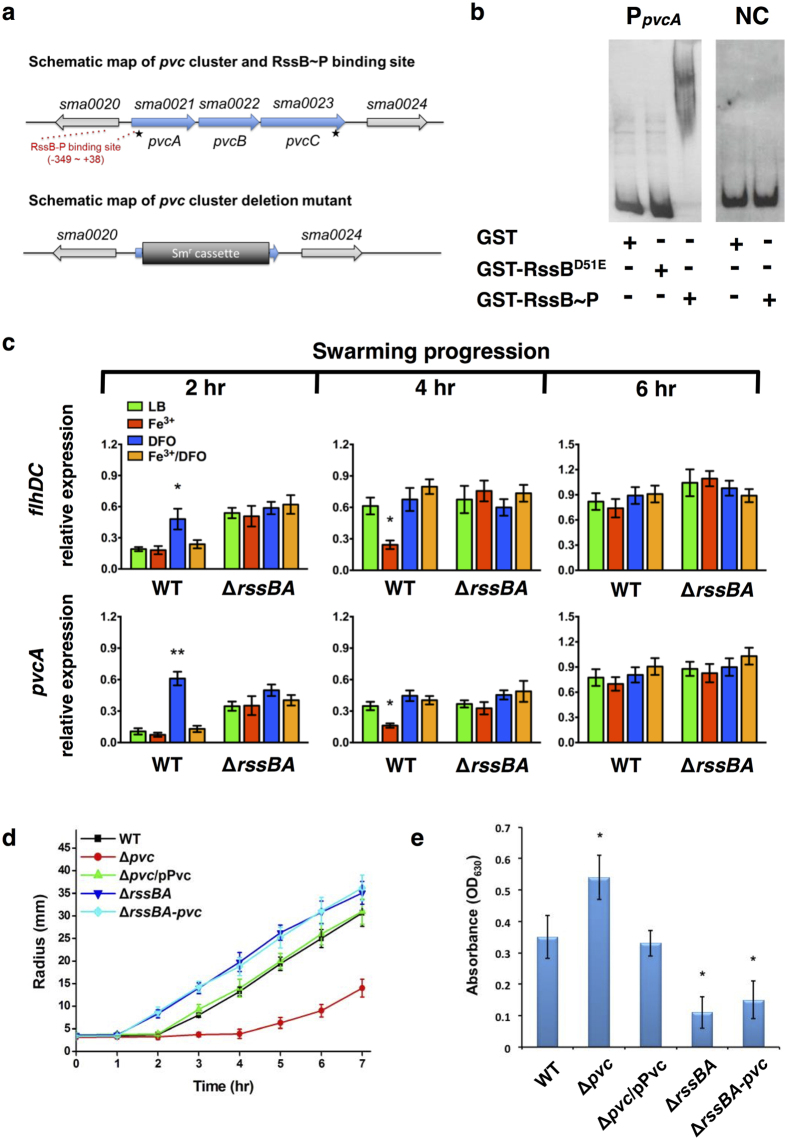
*pvc* cluster regulated by RssB is involved in regulating swarming and biofilm formation. **(a)** Schematic map of the *pvc* cluster, RssB-P binding site, and *pvc* cluster deletion mutant. Red dash lines represent RssB-P binding sites (−349 to +38) of the *pvcA* promoter region. For construction of *pvc* cluster deletion mutants (Δ*pvc*), genomic region between two asterisks (*) was replaced with Sm^r^ cassette. **(b)** EMSA was employed to confirm the interaction between phosphorylated RssB (RssB-P) and promoter region of *pvcA* (P_*pvcA*_). Digoxigenin (DIG)-labeled DNA fragments were incubated with purified GST, GST-RssB^D51E^ or GST-RssB-P, followed by analysis by non-denaturing PAGE. Negative control (NC) was performed by incubating GST-RssB-P with the DNA sequence between M13F/M13R in the plasmid pBIISK. **(c)** During swarming progression (2–6 hr) with different iron conditions, relative expression of RssB downstream genes (*flhDC* and *pvcA*), normalized to *16S rRNA*, in WT and Δ*rssBA* was respectively determined by qRT-PCR. **(d**,**e)** Swarming migration radius **(d)** and biofilm formation **(e)** of each strain of *S. marcescens*. Strain harboring pPvc encoding *pvc* cluster under pBAD promoter with 0.01% arabinose. The results shown represent means ± SEM from three independent experiments (n = 3). Statistical analysis was performed using one-way ANOVA. For Fig. 4c, * and ** corresponding to *P*-value < 0.05 and <0.01 in comparison with LB group of each strain, respectively. For Fig. 4e, * corresponding to *P*-value < 0.05 compared to WT.

To investigate whether RssAB signaling regulates extracellular Fe^3+^ availability, we performed an *in vitro* protein-DNA pull-down screening assay to identify genes involved in iron metabolism. We identified the promoter of the gene *sma0021*, annotated as *pvcA*, which is the first gene of the putative *pvc* cluster (Fig. 4a). The *pvc* cluster in *S. marcescens* is a homologue of the *pvc* operon in *Pseudomonas aeruginosa*, which was previously found to be involved in biosynthesis of pseudoverdine, a metabolite that possesses Fe^3+^ chelation activity^39^. Clarke-Pearson *et al*. observed that the *pvc* operon is responsible for the production of 2-isocyano-6,7-dihydroxycoumarin (ICDH-Coumarin), named by the authors as paerucumarin^40^,^41^, which regulates biofilm formation in *P. aeruginosa*^42^. Using an electrophoretic mobility shift assay (EMSA), we confirmed direct binding of the *pvcA* promoter to phosphorylated GST-RssB-P, instead of unphosphorylated GST-RssB^D51E^ or GST protein (Fig. 4b). We further showed that expression of *pvcA* in WT *S. marcescens* is down-regulated during the lag phase (2 hr), whereas it increases during the migration phase (4 hr) (Fig. 4c and Supplementary Fig. 7), in agreement with our previous observation that RssAB signaling is specifically activated in the lag phase to act as a transcriptional repressor (Fig. 2b). During swarming development, iron supplementation prolonged downregulation of the RssAB downstream genes *flhDC*^34^ and *pvcA* in an RssAB-dependent manner (Fig. 4c and Supplementary Fig. 7).

To understand the function of the *pvc* cluster in multicellular behavior, we constructed a whole *pvc* cluster deletion mutant (Fig. 4a). Compared to the WT strain, the *pvc* cluster deletion mutant (Δ*pvc*) showed delayed swarming initiation (Fig. 4d) and increased biofilm formation (Fig. 4e), with both processes being reversed by episomal expression of the *pvc* cluster (Fig. 4d,e; Δ*pvc*/pPvc). In contrast, the *rssBA* and *pvc* cluster double-deletion mutant exhibited early swarming initiation and reduced biofilm formation as observed in Δ*rssBA* (Fig. 4d,e; Δ*rssBA*-*pvc*). These data suggest that a metabolite produced by the *pvc* cluster may inhibit RssAB activation.

### The *pvc* cluster is responsible for ICDH-Coumarin production in *S. marcescens*

**Figure 5:**
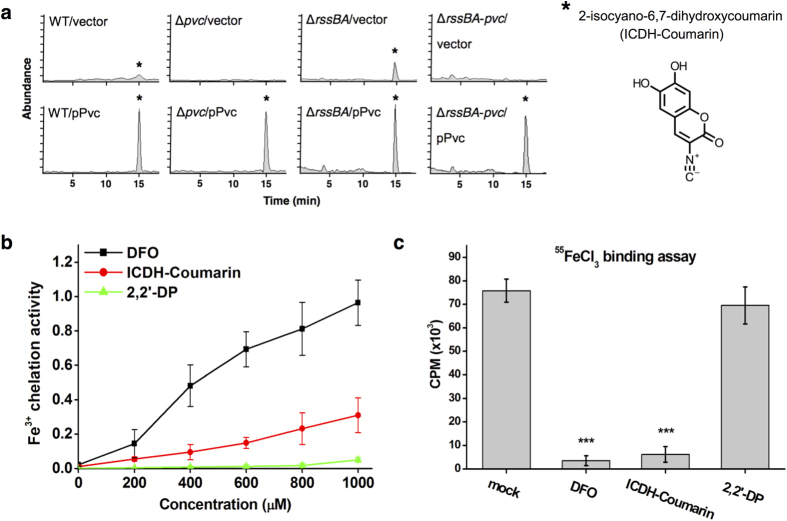
ICDH-Coumarin produced from *pvc* gene cluster chelates Fe^3+^ and blocks iron binding to RssA. **(a)** LC-MS chromatogram of ethyl acetate extracts from WT, Δ*pvc*, Δ*rssBA*, and Δ*rssBA*-*pvc* bacteria harboring pBAD33 (vector) or pPvc encoding *pvc* cluster under DMM broth with arabinose (0.3%). *Indicates the peak and structure of ICDH-Coumarin as determined by NMR. **(b)** Fe^3+^ chelation activity of DFO, ICDH-Coumarin, and 2,2′-DP at the indicated concentration. **(c)** His-tagged periplasmic domain of RssA was incubated with ^55^FeCl_3_ and distilled water (mock), DFO (0.3 mM), ICDH-Coumarin (0.3 mM), or 2,2′-DP (0.3 mM). Radioactivity was determined from the eluted periplasmic domain. One-way ANOVA with *** corresponding to *P*-value < 0.001 compared to mock group.

To determine whether the *pvc* cluster in *S. marcescens* is responsible for the production of a molecule similar to pseudoverdine or paerucumarin identified in *P. aeruginosa*, we used liquid chromatography-mass spectrometry (LC-MS) (Fig. 5a, Supplementary Fig. 8a) and nuclear magnetic resonance (NMR) (Supplementary Fig. 8b) to identify the compounds that were enriched in *S. marcescens* over-expressing the *pvc* cluster (pPvc). A compound corresponding to 2-isocyano-6,7-dihydroxycoumarin (ICDH-Coumarin) (with the same structure as paerucumarin) was identified (Fig. 5a; highlighted as *; Supplementary Fig. 8)^40^; the ICDH-Coumarin compound was not observed in Δ*pvc* bacteria expressing the vector only. The purified ICDH-Coumarin harbored Fe^3+^ chelation activity similar to that of DFO but to a lesser extent (Fig. 5b). ICDH-Coumarin prevented direct binding of Fe^3+^ to the periplasmic domain of RssA (Fig. 5c). These findings demonstrate that the *pvc* cluster is implicated in ICDH-Coumarin production and that ICDH-Coumarin can chelate Fe^3+^ to abolish RssA-Fe^3+^ binding.

### ICDH-Coumarin controls RssAB signaling and multicellular behaviors by modulating extracellular Fe^3+^ availability

**Figure 6:**
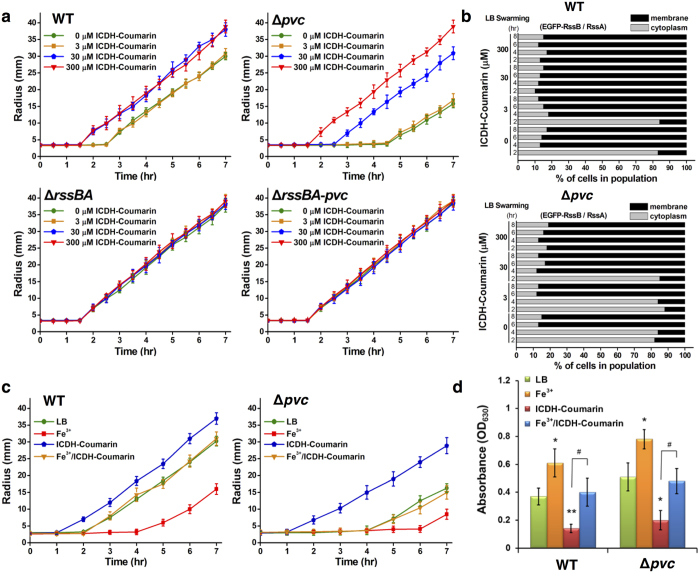
ICDH-Coumarin induces swarming initiation and represses biofilm formation by modulating extracellular Fe^3+^ availability and RssAB signaling. (**a**) Migration radius of WT, Δ*rssBA*, Δ*pvc*, and Δ*rssBA*-*pvc* strains on LB swarming plates supplemented with ICDH-Coumarin (0–300 μM). (**b**) Quantified RssAB signaling of WT and Δ*pvc* carrying pEGFP-RssBA::Gm during swarming progression (2–8 hr) on LB swarming plates containing arabinose (0.1%) and ICDH-Coumarin (0–300 μM) is shown. (**c**) Migration radius of WT and Δ*pvc* on LB swarming plates supplemented with or without Fe^3+^ (100 μM) and ICDH-Coumarin (300 μM). (**d**) Biofilm of WT and Δ*pvc* in LB condition supplemented with or without Fe^3+^ (100 μM) and ICDH-Coumarin (300 μM) was determined by monitoring absorbance at 630 nm. The results shown represent means ± SEM from three independent experiments (n = 3). One-way ANOVA with * and ** represent *P* values < 0.05 and <0.01 compared to LB group; ^#^ represents *P*-value < 0.05 compared to ICDH-Coumarin group.

We aimed to determine whether ICDH-Coumarin might alter extracellular iron availability and subsequently regulate RssAB signaling as well as multicellular behaviors. While addition of 30 μM ICDH-Coumarin restored the delayed swarming migration phenotype of Δ*pvc* and induced early swarming migration in WT *S. marcescens* similar to DFO (Fig. 6a; 30 μM), supplementation of 300 μM ICDH-Coumarin induced early swarming migration even in Δ*pvc* bacteria (Fig. 6a; 300 μM ICDH-Coumarin for Δ*pvc*). Moreover, early swarming initiation induced by ICDH-Coumarin was accompanied by deactivation of RssAB signaling in both WT and Δ*pvc* bacteria (Fig. 6b). The effects of ICDH-Coumarin supplementation on swarming initiation and RssAB signaling could also be observed by overexpression of the *pvc* cluster (Supplementary Fig. 9a,b). Conversely, regulation of swarming initiation by ICDH-Coumarin or *pvc* overexpression was completely abolished in the absence of *rssBA* (Fig. 6a and Supplementary Fig. 9a,b). On the other hand, addition of ICDH-Coumarin induced early swarming initiation (Fig. 6c) and impaired biofilm formation similar to DFO treatment (Fig. 6d), and these effects could be restored by addtion of Fe^3+^. Taken together, our results demonstrate that ICDH-Coumarin is produced by the RssAB-regulated *pvc* cluster and that it regulates swarming and biofilm formation by altering extracellular Fe^3+^ availability and RssAB signaling.

## Discussion

**Figure 7:**
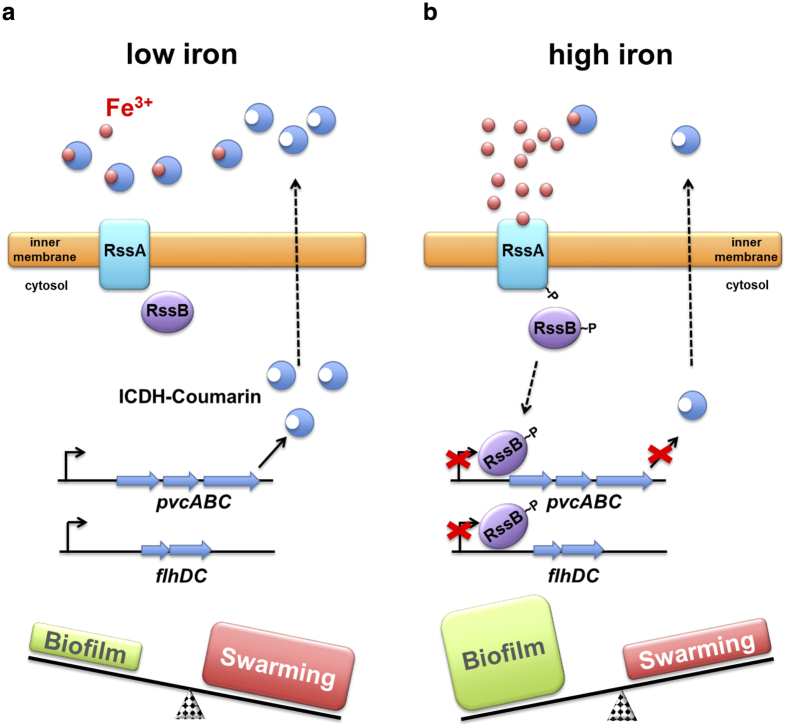
Proposed model for the control of swarming and biofilm formation by the RssAB-ICDH-Coumarin-iron pathway. **(a)** Low extracellular Fe^3+^ deactivates RssAB signaling and relieves transcription of the *pvc* cluster and *flhDC*, which encodes the master regulator of flagellum biosynthesis This in turn increases flagellum and production of ICDH-Coumarin which subsequently chelates Fe^3+^ to maintain low availability of free Fe^3+^. These processes induce swarming and repress biofilm formation. **(b)** High extracellular Fe^3+^ activates RssAB signaling, leading to phosphorylation of RssB, repression of *pvc* cluster *and flhDC* transcription, and reduced ICDH-Coumarin and flagellum production. High Fe^3+^ availability sustains activation of RssAB signaling. These processes lead to biofilm formation and inhibit swarming migration by restraining bacteria in the swarming lag phase.

Swarming and biofilm formation are two opposite but inter-related bacterial behaviors that are also among the most ancient features of living cells^10^. Here, we demonstrate that environmental Fe^3+^ availability controls the transition between swarming initiation and biofilm formation through an RssAB signaling system in *S. marcescens*. We further determine that the RssAB-modulated *pvc* cluster produces the Fe^3+^ chelator ICDH-Coumarin to regulate extracellular iron availability and RssAB signaling (Fig. 7). Our results show that RssAB signaling is off at low Fe^3+^ concentrations, during which the *pvc* cluster is expressed to produce ICDH-Coumarin and chelate extracellular Fe^3+^ (Fig. 7a). In an environment in which Fe^3+^ is abundant, Fe^3+^ directly binds to RssA, leading to RssA autophosphorylation and RssAB transphosphorylation, resulting in downregulated expression of the *pvc* cluster, reduced ICDH-Coumarin production, and decreased extracellular Fe^3+^ chelation (Fig. 7b).

Bacteria utilize a broad array of strategies to control the timing and duration of TCS signaling events in order to precisely control cellular processes based on extracellular signals^43^. In the context of the RssAB-ICDH-Coumarin-iron regulation circuit (Fig. 7), *S. marcescens* actively regulates extracellular iron availability through RssAB-modulated production of ICDH-Coumarin. Upon sensing high extracellular Fe^3+^ concentration, the decrease in ICDH-Coumarin production by transcriptional repression of phosphorylated RssB in turn maintains active RssAB signaling, which restricts bacterial migration and promotes biofilm formation. Of note, we previously reported that RssAB activation suppresses bacterial swarming motility by repressing *flhDC* expression^34^, whereas overexpression of *flhDC* reduces biofilm formation^33^. Together with the results presented in this study that functional RssAB signaling is required for iron to downregulate *flhDC* expression, restrict swarming migration and promote biofilm formation, we highlight the pivotal role of iron-RssAB-FlhDC signaling in regulation of swarming and biofilm formation (Fig. 7). These results also indicate that tight regulation of flagellum production by RssAB-ICDH-Coumarin-iron is crucial for the development of multicellular behavior in *S. marcescens*.

Based on LC-MS and NMR analyses, ICDH-Coumarin secreted by *S. marcescens* (Fig. 5 and Supplementary Fig. 8) and identified in this study has the same molecular structure (2-isocyano-6,7-dihydroxycoumarin) as paerucumarin in *P. aeruginosa*^40^,^41^. While ICDH-Coumarin (paerucumarin) enhances biofilm formation in *P. aeruginosa* by upregulating the fimbrial synthesis pathway^42^, we demonstrated here that ICDH-Coumarin reduces biofilm formation in *S. marcescens*. The different roles of this iron-chelating molecule in controlling multicellular behavior in *P. aeruginosa* and *S. marcescens* indicate that different cellular machineries may have evolved in response to a specific extracellular signal. Conservation of the *pvc* cluster across various bacterial species^40^ and the function of ICDH-Coumarin in regulating bacterial behavior suggest that ICDH-Coumarin may be involved in interspecies communication.

Competition for iron between microbes in the environment usually involves the coordination of various bacterial activities, including oxidative stress response, antibiotics resistance, virulence and multicellular behavior^44^,^45^,^46^,^47^. Previously, the TCS PmrA-PmrB was found to be vital for survival of *Salmonella enterica* under high iron stress through direct sensing of extracellular iron^29^. It was further shown that high iron resistance is mediated by lipopolysaccharide modifications^48^. Here we show that RssAB participates in a sophisticated control system to regulate multicellular behavior without conferring iron resistance since *rssBA* deletion does not affect growth in either iron-abundant or iron-limiting conditions. The presence of multiple sensing systems in deciphering iron availability may provide flexibility for bacteria to thrive under changing environments. In summary, this study identifies a cellular mechanism underlying the transition between bacterial motility and static colonization, which are associated with acute and chronic infection, respectively, in response to extracellular iron availability. Our findings should prove helpful to understand the factors that determine bacterial acute or chronic infection as well as for the development of novel treatments against pathogenic bacteria.

## Methods

### Bacterial strains and culture conditions

*S. marcescens* strains were derived from *S. marcescens* CH-1 (WT). Bacteria were routinely cultured with agitation in LB broth (BD Difco^TM^, U.S.A.) at 30 °C or 37 °C. M9 salt (BD Difco^TM^, U.S.A.) solution^49^ was used to make defined minimal medium (DMM) containing 1× M9 salts, 2 mM magnesium sulfate, 100 μM calcium chloride, 0.8% glycerol, and 0.2% casamino acid. DMM was used as iron-limiting medium. When strains harboring pBAD series of plasmids were used, arabinose was added into the medium at the indicated concentrations. Bacterial strains and plasmids are summarized in Supplementary Table 1 and Supplementary Methods.

### Swarming assay

Swarming assay was performed on swarming plates in the presence or absence of metal ions, metal chelators, ICDH-Coumarin, or reducing agent at the indicated concentrations. *S. marcescens* strains were cultured on swarming plates (0.8% Eiken agar, EIKEN Chemical, Japan) consisting of LB (BD Difco™, U.S.A.) or DMM medium. The swarming lag phase was defined as the static period prior to migration.

### Biofilm assay

Biofilm formation assay was performed based on a previous study^35^. Briefly, overnight cultures were diluted 1:100 in LB medium containing 1% (w/v) sucrose in Petri dish with sterile glass coverslips for incubation at 30 °C with agitation at 50 rpm. For iron modification, 10-hr-old biofilm cultures were supplemented with ferric chloride (Sigma-Aldrich, U.S.A.), DFO (final concentration: 0.3 mM) (Sigma-Aldrich, U.S.A.) and/or ICDH-Coumarin at the indicated concentration. Biofilm mass was quantified on glass coverslips at maturation stage (24 hr) using crystal-violet staining and spectrophotometery detection at 630 nm (OD_630_). Results are shown as means ± standard error of the mean (SEM) based on three independent experiments.

### Imaging and quantification of RssAB signaling using fluorescence microscopy

RssAB signaling was determined by localization of EGFP-tagged RssB as before^35^. Briefly, EGFP-RssB and RssA either from pEGFP-RssBA::Sm^35^ or pEGFP-RssBA::Gm (Supplementary Table 2) were co-expressed under the P_BAD_ promoter control and induced by 0.1% arabinose. At the indicated time points of swarming and biofilm assay, cells were harvested to determine the percentage of population showing EGFP-RssB localized at the cell membrane (OFF) or in the cytoplasm (ON). Fluorescence microscopy was conducted with a Leica DM2500 microscope under a Leica I3 filter set and observed at 100x using an oil immersion objective. Images were taken with a SPOT RT3 CCD camera (Diagnostic Instruments, U.S.A) and adjusted using the SPOT Advanced software (Diagnostic Instruments, U.S.A.). At least 200 cells were counted for each assay condition. Results are shown as the average of percentage of two populations from three independent experiments.

### Liposome-based phosphorelay of RssAB signaling cascade

Liposome-based resconstitution^38^ of purified RssA and RssB, either untreated or supplemented with the indicated concentration of iron, reducing agent, or iron chelators, was subjected to radioisotope [γ^32^P]ATP (PerkinElmer, U.S.A.) phosphorelay.

### Iron binding assay

Purified His-tagged RssA periplasmic domain was incubated with 500 nM ^55^FeCl_3_ and 0.3 mM of DFO, 2,2′-DP (Sigma-Aldrich, U.S.A.), ASC (Sigma-Aldrich, U.S.A.), or ICDH-Coumarin. After passing through Ni^2+^-nitrilotriacetic acid (NTA) affinity chromatography (GE Healthcare Lifesciences, U.S.A.), the protein was eluted and subjected to liquid scintillation counting to measure radioactivity of ^55^Fe, expressed as counts per min (CPM × 10^3^). For His-tagged membrane proteins, including RssA, RssA^H248A^, RssA^ΔPPD^, and RssA^chimeric^, each of them was reconstituted in liposome containing 500 nM ^55^FeCl_3_, followed by removal of unbound ^55^FeCl_3_ and elution of iron using NTA column. The resulting eluents were subjected to liquid scintillation counting.

### *In vitro* protein-DNA pull-down assay

The assay used was modified from Dietz *et al*.^50^. Briefly, WT *S. marcescens* chromosomal DNA was digested by *Sau*3AI and resuspended in 1 ml of interaction buffer (20 mM Tris-HCl, pH 7.5, 10 mM MgCl_2_, 100 mM KCl) containing 25 mM acetyl-phosphate, 5 mM EDTA and 10 μg/ml BSA. GST-RssB was phosphorylated by 50 mM acetyl-phosphate at 37 °C for 1 hr, prior to addition into the mixture containing WT *S. marcescens* chromosomal DNA fragments. After incubation at room temperature for 20 min, 30 μL glutathione sepharose-4B beads equilibrated with PBS were added into the mixture. The whole mixture was placed at 4 °C with constant shaking for 30 min. The beads bound to GST-RssB with binding DNA fragments were recovered by low-speed centrifugation. After three washing steps with 500 μl of interaction buffer, the DNA was purified by phenol extraction and precipitated with isopropanol. Following precipitation, the bound DNA was analyzed on 2% agarose gel, and cloned into the *Bam*HI site of pBluscriptIISK. The GenBank accession number of *pvcABC* is KC291199.

### Electrophoretic mobility shift assay (EMSA)

Promoter region of *pvcA* (P_*pvcA*_) and *rssB* (P_*rssB*_) was cloned into the BamHI site on pBluescript II SK+ to generate pBSK-P_*pvcA*_ and pBSK-P_*rssB*,_ respectively. DNA fragments for electrophoretic mobility shift assay were amplified by PCR using the M13F-DIG/M13R primer pairs using pBSK-P_*pvcA*_, pBSK-P_*rssB*_ or pBluescript II SK+ (negative control, NC) as a template. GST, GST-RssB and GST-RssB^D51E^ protein purification, and GST-RssB phosphorylation using acetyl-phosphate (Sigma-Aldrich, U.S.A.) were performed as described in our previous report^35^. Phosphorylated GST-RssB or GST-RssB^D51E^ was diluted in binding reaction buffer (20 mM Tris-HCl, pH 7.5, 10 mM MgCl_2_, 100 mM KCl) before binding assay. The binding reaction was performed in binding reaction buffer, comprising the protein as indicated and 0.5 ng DIG labeled DNA fragments supplemented with 30 μg/ml poly (dI-dC) and 1 μg/μl bovine serum albumin. The reaction mixtures were incubated for 30 min at room temperature before being loaded onto 6% nondenaturing polyacrylamide gels containing 0.5× TBE buffer. Electrophoresis was performed at 100 V for 1–4 hr. The DNA-protein complexes were then electroblotted onto a positively charged Hybond-N nylon membrane (Amersham, U.S.A.) and detected using alkaline phosphatase conjugated anti-DIG antibodies (Roche Life Science, U.S.A.). CSPD (Roche Life Science, U.S.A.) was used for substrate as described by the manufacturer. Membranes were exposed to X-ray film at room temperature for 2 to 30 min.

### Evaluation of gene expression

Total bacterial RNA was extracted using a Trizol kit (Invitrogen). After verifying the quality (A260/A280 = 1.8–2.0) and concentration, 200 ng of RNA was subjected to reverse-transcription into cDNA with a SuperScript III First-Strand Synthesis System kit (Invitrogen, U.S.A.) according to the manufacturer’s instructions. 5 ng of cDNA was then applied to KAPA SYBR FAST Master Mix (2X) qPCR kit (Kapa Biosystems, South Africa). Expression level of target genes tested was verified by real-time quantitative PCR detection system (Roche LightCycler 480, U.S.A.). Melting curves and Ct values were analyzed using the LightCycler^®^ 480 SW version 1.5 (Roche, U.S.A.). The data were analyzed using the 2^−ΔΔC^T method^51^. Relative expression of target genes was normalized to *16S rRNA* (Fig. 4c) or *rpoD* (Supplementary Fig. 7). The procedures used for qRT-PCR followed the MIQE guidelines^52^. The primers used in this study are summarized in Supplementary Table 2.

### Detection and purification of 2-isocyano-6,7-dihydroxycoumarin (ICDH-Coumarin)

ICDH-Coumarin was detected and purified according to the procedures described previously^40^ with minor modifications. Briefly, *S. marcescens* harboring pBAD33 (vector) or pPvc containing the full-length *pvc* cluster was grown in DMM broth with arabinose (0.3%) at 30 °C for 8 hr. Ethyl acetate extraction of each supernatant was collected by mixing the supernatant (50 ml) with ethyl acetate containing 2% methanol (100 ml). ICDH-Coumarin from ethyl acetate extract was purified using silica gel flash columns (CHCl_3_:MeOH at 9:1 followed by CH_2_Cl_2_:MeOH:AcOH at 93:7:0.1). The resultant filtrate was applied to LC-MS (negative-ion electrospray ionization, Waters, U.S.A.) and NMR (Bruker Avance III-HD 600 MHz NMR Spectrometer, U.S.A.). For LC-MS, 25 μl of a 10-fold dilution of the resultant filtrate was subjected to LC-MS (XBridge C18 column of 5 μm, 4.6×100 mm). UV absorbance from 210 to 600 nm was measured on a Waters Photodiode Array Detector. Spectrum of ESI-LC-MS was plotted as intensity (y axis) against m/z (Da). The m/z of ICDH-Coumarin is 204.03023. For NMR conditions, vacuum-dried samples were dissolved in 600 μl of methanol-d4/dichloromethane-d2 (CD_3_OD/CD_2_Cl_2_), followed by vortexing (1 min), sonication (5 min), and vortexing (1 min). After centrifugation (13,200 rpm), the supernatant was transferred to a NMR tube (5 mm). NMR spectra were recorded at 600 MHz on a Bruker Avance III spectrometer. 1H-NMR (CD_3_OD/CD_2_Cl_2_) proton spectrum of ICDH-Coumarin was ploted as intensity (y axis) against chemical shift (x axis) (δ, ppm). The identified chemical shift of ICDH-Coumarin includes 9.50 (s, 1H), 8.39 (s, 1H), 7.67 (s, 1H), 6.87 (s, 1H), and 6.87 (s, 1H).

### Determination of Fe^3+^ chelation activity

Fe^3+^ chelation activity was determined using the chrome azurol sulfonate (CAS, Sigma-Aldrich, U.S.A.) solution assay^53^ with minor modifications. Briefly, 100 μl of ICDH-Coumarin (in ethyl acetate), DFO (in water), and 2,2′-DP (in dimethyl sulfoxide) at the indicative concentration (μm) or the respective solvent (as blank) were mixed with 100 μl of CAS solution and 10 μl of 0.2 M 5′-sulfosalicylic acid, prior to incubation at room temperature for 15 min. Absorbance at a wavelength of 630 nm for sample (A_s_) and blank (A_b_) was determined using a spectrophotometer. Fe^3+^ chelation activity was expressed as the ratio of (A_b_ − A_s_)/A_b_.

### Statistical analysis

Data were expressed as means ± SEM from three independent experiments (n = 3). Statistical difference was calculated by one-way analysis of variance (ANOVA) by comparing the groups indicated. *P*-value < 0.05 was considered statistically significant.

## Additional Information

**How to cite this article**: Lin, C. S. *et al*. An iron detection system determines bacterial swarming initiation and biofilm formation. *Sci. Rep.* **6**, 36747; doi: 10.1038/srep36747 (2016).

**Publisher’s note:** Springer Nature remains neutral with regard to jurisdictional claims in published maps and institutional affiliations.

## Supplementary Material

Supplementary Information
