# Supplementary material for: An iron detection system determines bacterial swarming initiation and biofilm formation
Source: Sci Rep. 2016 Nov 15;6:36747. doi: 10.1038/srep36747 (PMC5109203; doi:10.1038/srep36747)
Supplement: Supplementary Information [file srep36747-s1.pdf]

Supplementary Information

An iron detection system determines bacterial swarming initiation and biofilm formation

Chuan-Sheng Lin, Yu-Huan Tsai, Chih-Jung Chang, Shun-Fu Tseng, Tsung-Ru Wu, Chia-Chen Lu, Ting-Shu Wu, Jang-Jih Lu, Jim-Tong Horng, Jan Martel, David M. Ojcius, Hsin-Chih Lai, and John D. Young

This Supplementary Information includes Supplementary Figures 1-9, Supplementary Tables 1-2, Supplementary Methods, and Supplementary References.

Supplementary Figures

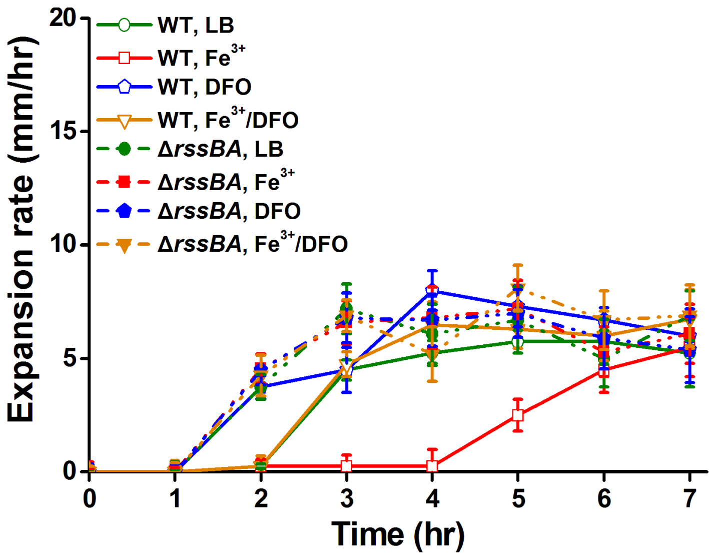

Supplementary Figure 1. Fe^{3+} regulates swarming initiation timing without affecting swarming expansion rate. Swarming expansion rate (mm/hr) of WT and Δ*rssBA S. marcescens* was measured on LB swarming plates containing Fe^{3+} (100 μM) and/or DFO (0.3 mM). The conditions used here are the same as Fig. 1a,b.

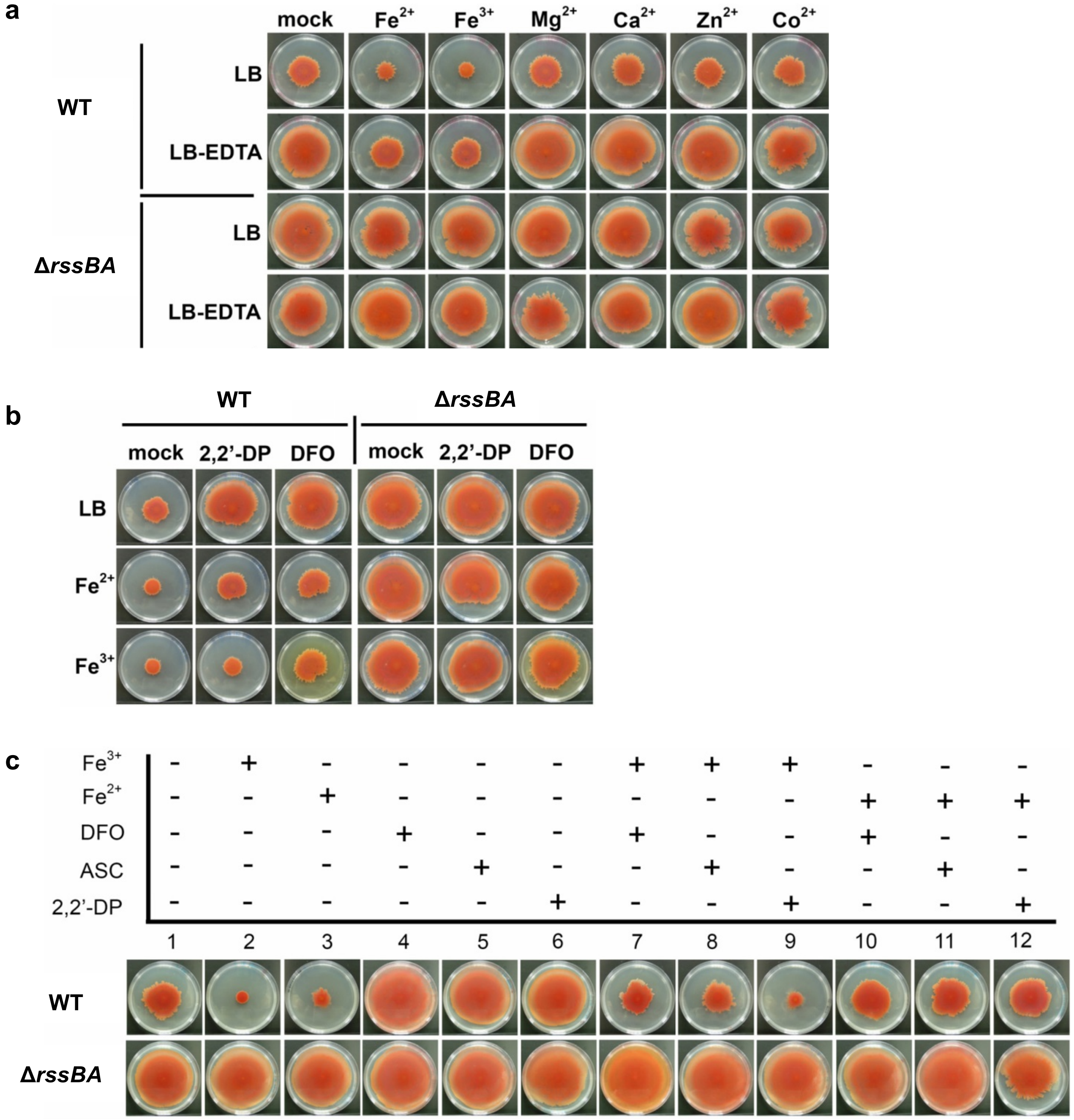

Supplementary Figure 2. RssAB represses swarming behavior in response to free Fe^{3+}. (a) Swarming of WT and Δ*rssBA* bacteria was assessed on LB swarming plates in the presence or absence of various metal ions including $FeSO_4$ (Fe^{2+}), $FeCl_3$ (Fe^{3+}), $MgSO_4$ (Mg^{2+}), $CaCl_2$ (Ca^{2+}), $ZnSO_4$ (Zn^{2+}), and $CoCl_2$ (Co^{2+}) at 100 μM, with/without metal ion chelator (EDTA, pH 7.4, 0.3 mM). **(b)** Swarming of WT and Δ*rssBA* bacteria on LB swarming plates in the presence or absence of Fe^{2+} (100 μM) and Fe^{3+} (100 μM) with/without chelators (2,2'-DP or DFO, 0.3 mM). **(c)** Swarming of WT and Δ*rssBA* bacteria on LB swarming plates in the presence of iron under various conditions. Fe^{3+}, 100 μM; Fe^{2+}, 100 μM; DFO, 0.3 mM; 2,2'-DP, 0.3 mM; ASC, 0.3 mM. Mock, distilled water. Representative swarming plates of three independent experiments are shown.

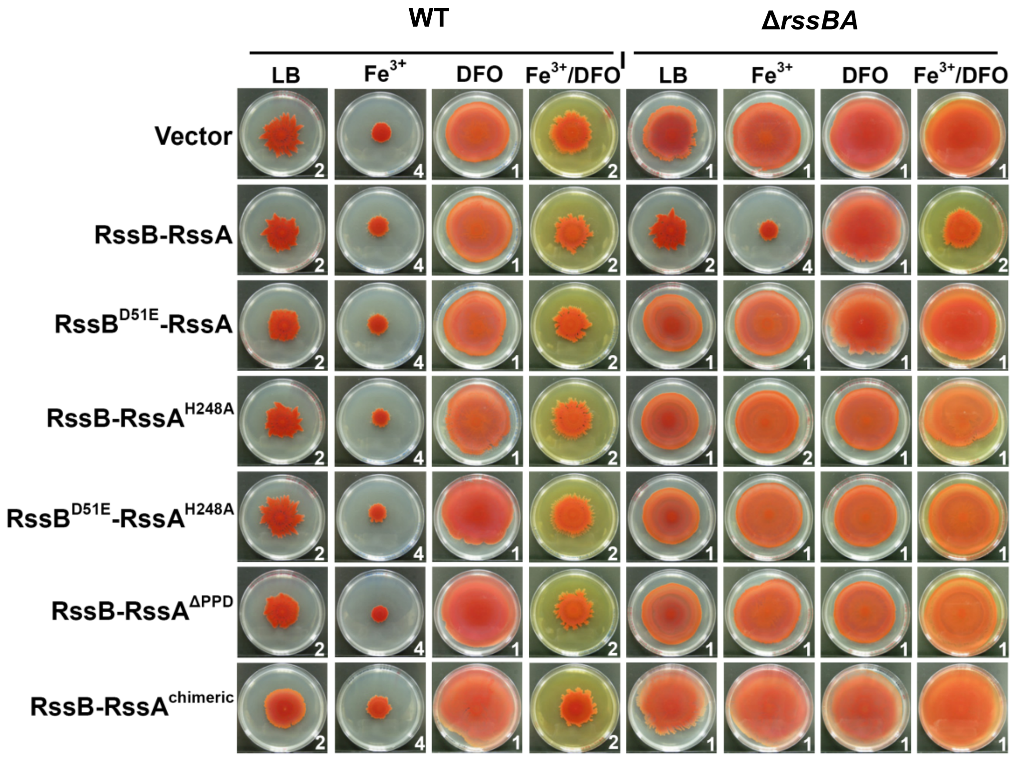

Supplementary Figure 3. RssAB phosphotransfer signaling is required for Fe^{3+}-regulated swarming initiation in *S. marcescens*. Swarming of WT and Δ*rssBA* bacteria harboring the empty vector or the recombinant pACYC184 plasmid encoding different constructs of RssB and RssA driven by their own promoter was monitored. Swarming was evaluated on LB swarming plates with or without Fe^{3+} (100 μM) and DFO (0.3 mM). $RssB^{D51E}$ and $RssA^{H248A}$: constructs with mutations in conserved phosphorylation sites; $RssA^{\Delta PPD}$: RssA with deletion in periplasmic domain; $RssA^{chimeric}$: chimeric RssA whose periplasmic domain was replaced with the periplasmic domain of QseC. Representative swarming plates of three independent experiments are shown. The number in the bottom right corner of each swarming plate image represents the duration of the lag phase in hours. The migration radius of Δ*rssBA* is shown in Fig. 1e.

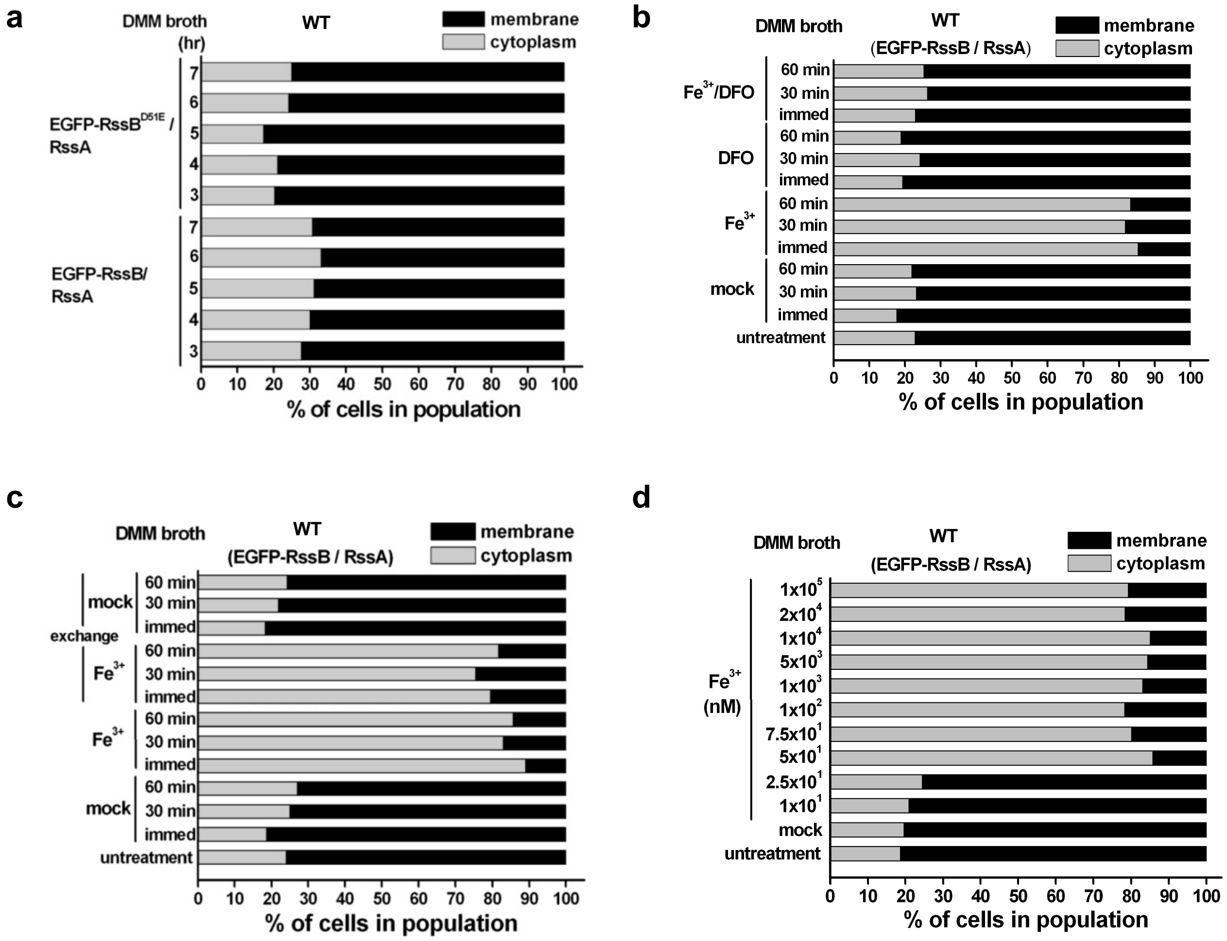

Supplementary Figure 4. Fe^{3+} rapidly alters RssAB signaling at the nanomolar level. (a) *S. marcescens* WT harboring either pEGFP-RssBA::Sm or pEGFP-RssB^{D51E}-RssA::Sm (as a control of inactive RssAB signaling) was cultivated in the iron-limited DMM broth containing 0.1% arabinose for 3 to 7 hrs, and RssAB signaling was monitored. **(b)** After cultivation for 5 hrs, broth media were either left untreated (untreatment) or treated with mock sterile water, Fe^{3+} (100 μM), DFO (0.3 mM), or Fe^{3+}/DFO. RssAB signaling was then monitored immediately (immed) or 30 min and 60 min after addition. **(c)** After cultivation 5 hrs, the media were then supplemented with mock water or Fe^{3+} (100 μM), followed by cultivation for the time indicated. Supernatant in the mock group was split as untreated or interchanged with that from Fe^{3+} culture at each time point, followed by monitoring RssAB signaling status. **(d)** After cultivation for 5 hrs, the culture broth was either left untreated (untreatment) or supplemented with mock water or Fe^{3+} at the indicated concentrations (10 nM–100 μM). RssAB signalling was monitored immediately (immed) after supplementation. The culture broth used here corresponds to DMM broth containing 0.1% arabinose. Percentage of cell type was determined as the means of three independent experiments (n=3).

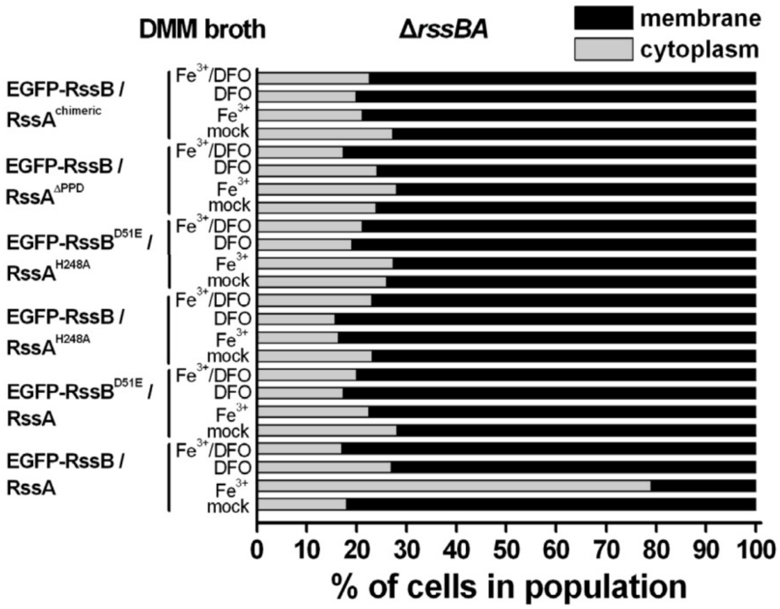

Supplementary Figure 5. Functional RssAB phosphotransfer is essential for Fe^{3+} responsiveness in RssAB signaling. *S. marcescens* Δ*rssBA* harboring recombinant pBAD24 encoding different forms of EGFP-RssB and RssA was cultivated in DMM broth containing 0.1% arabinose for 5 hrs, followed by supplementation with mock water, Fe^{3+} (LB: 100 μM, DMM: 10 μM), DFO (0.3 mM) or Fe^{3+}/DFO. RssAB signaling was then monitored. Percentage of cell type was determined as the means of three independent experiments (n=3).

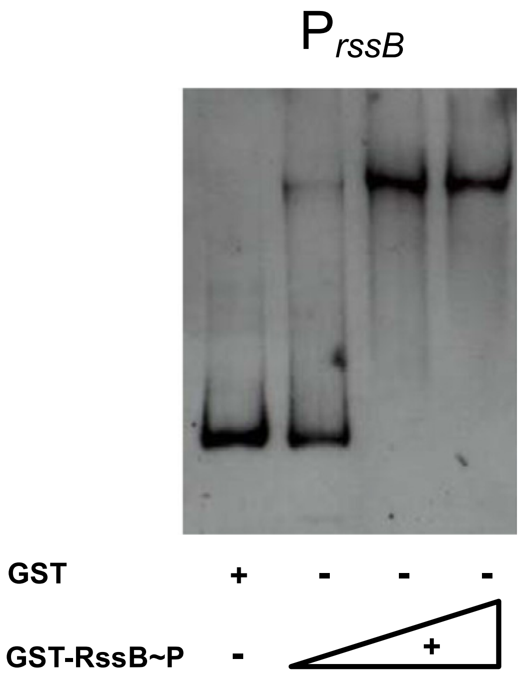

Supplementary Figure 6. Phosphorylated RssB binds to the promoter region of *rssB*. The promoter region of *rssB* (P_{rssB}) was used as a positive control for phosphorylated RssB-bound fragment in EMSA. GST (4μM) or GST-RssB~P (0.1, 0.5 or 1μM) was used.

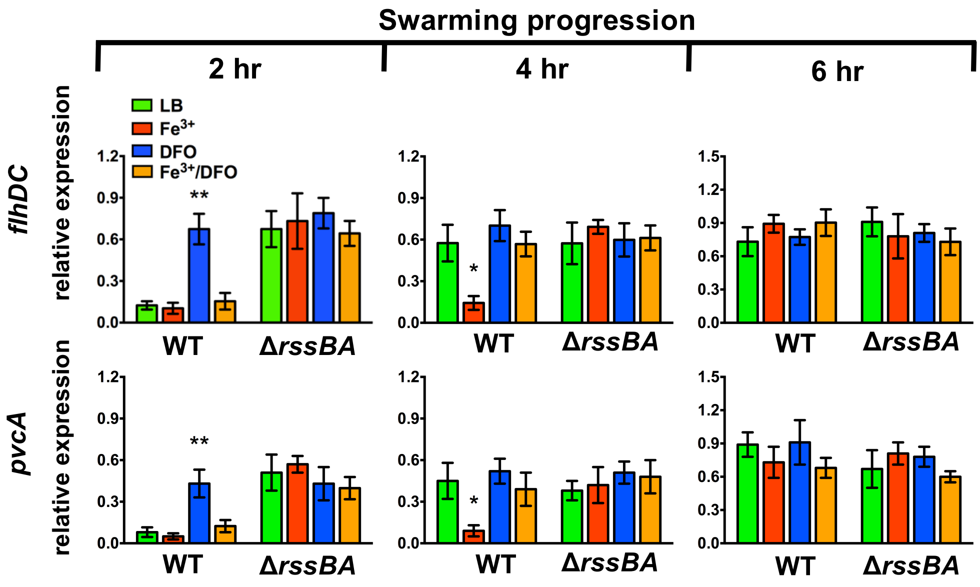

Supplementary Figure 7. Fe^{3+} modulates RssB-regulated downstream genes in swarming development. During swarming progression (2–6 hrs) and under different iron conditions, relative expression of RssB downstream genes (*flhDC* and *pvcA*) was assessed by qRT-PCR in WT and Δ*rssBA* bacteria. Expression was normalized to *rpoD*. Statistical analysis was performed using one-way ANOVA, with * and ** corresponding to *P*-values <0.05 and <0.01 in comparison with the LB group of each strain.

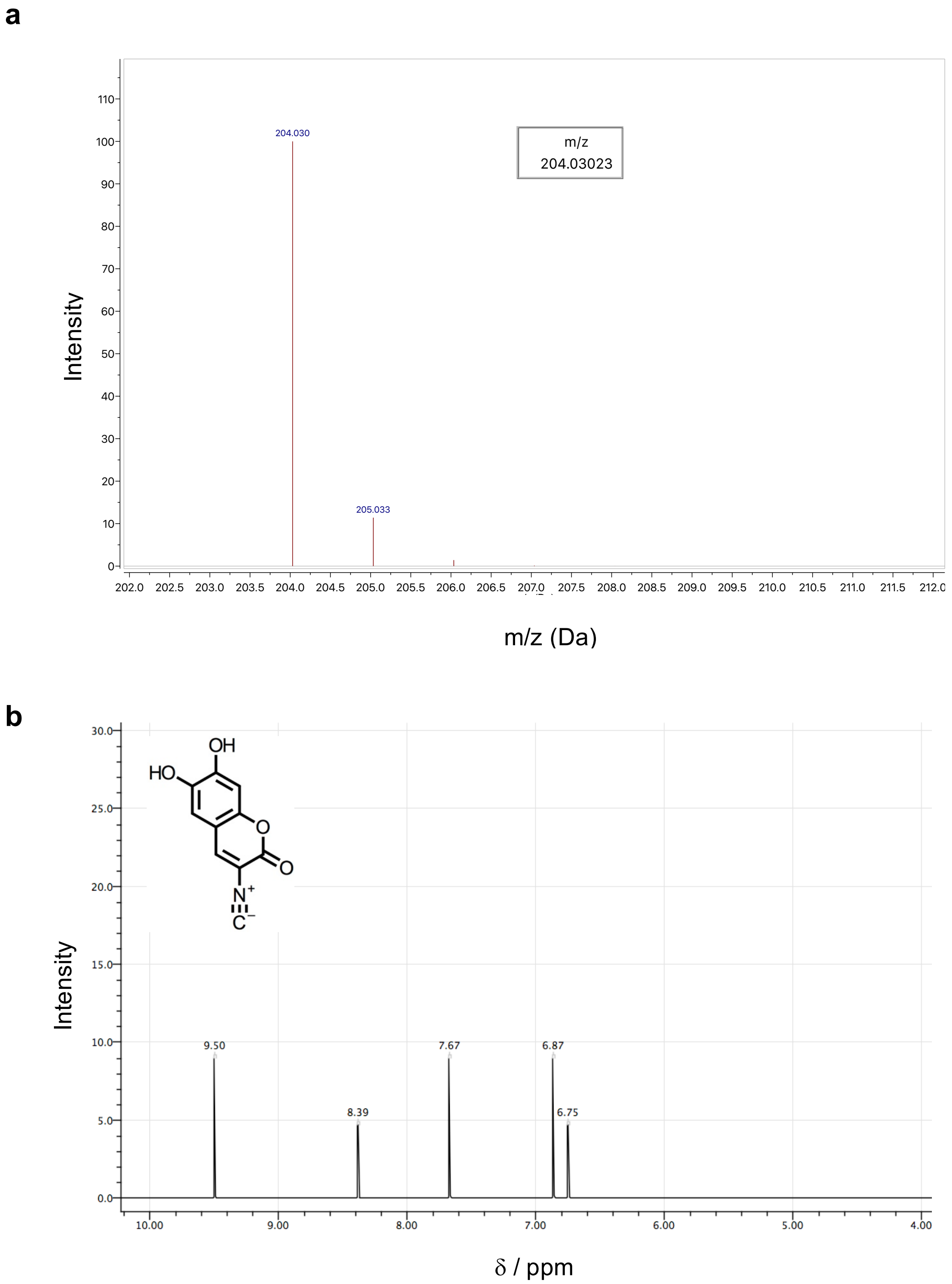

Supplementary Figure 8. LC-MS and NMR spectra of ICDH-Coumarin. (a) Mass spectrum of ESI-LC-MS shows signal intensity (y axis) against m/z (Da) (x axis). The m/z of ICDH-Coumarin is m/z: 204.03023. **(b)** 1H-NMR (CD_3OD/CD_2Cl_2) proton spectrum of ICDH-Coumarin shows signal intensity (y axis) against chemical shift (x axis; δ/ppm). The identified chemical shifts of ICDH-Coumarin include 9.50 (s, 1H), 8.39 (s, 1H), 7.67 (s, 1H), 6.87 (s, 1H), and 6.75 (s, 1H). The chemical structure of ICDH-Coumarin is shown.

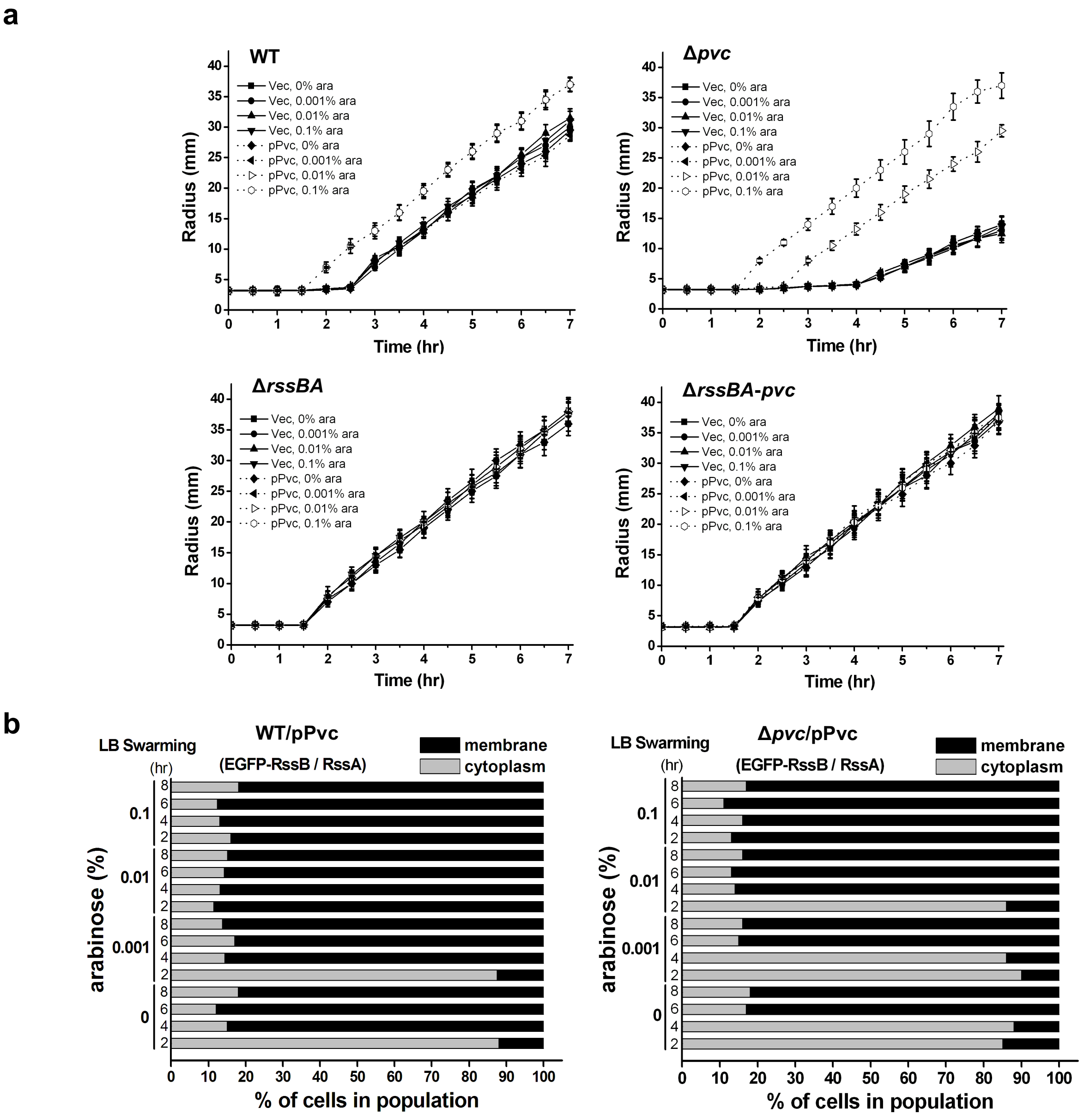

Supplementary Figure 9. Ectopic expression of *pvcABC* induces swarming initiation by repressing RssAB signaling. (a) Swarming radius of *S. marcescens* strains harboring either pBAD33 (vector) or pPvc encoding the *pvc* cluster driven by the P_{BAD} promoter was measured on LB swarming plates containing arabinose (0–0.1%). **(b)** Quantification of RssAB signaling for WT and Δ*pvc* carrying pPvc and pEGFP-RssBA::Gm during swarming progression (2–8 hrs) on LB swarming plate containing arabinose (0–0.1%).

Supplementary Tables

Supplementary Table 1. Bacterial strains and plasmids used in this study

Strain or Plasmid	Genotype or relevant characteristics	Source or reference
E. coli		
DH5α	F^-, φ80d*lacZ*ΔM15, Δ(*lacZYA-argF*)U169, *deoR*, *recA*1, *endA*1, *hsdR*17(rk^-, mk^+), *phoA*, *supE*44, λ^-, *thi*-1, *gyrA*96, *relA*1	Invitrogen
CC118	λ-*pir* lysogen [Δ(*ara-leu*) *araD* Δ*lacX74 galE galK phoA20 thi-1 rpsE rpoB argE*(Am) *recA1*]; permissive host for suicide plasmids requiring Pir protein	[1]
S17-1	λ-*pir* lysogen [*thi pro hsdR hsdM*$^+$ *recA* RP4 2-Tc::Mu-Km::Tn*7* (Tp^r Sm^r)]; permissive host able to transfer suicide plasmid pDM4 to recipient cells via conjugation	[1]
C41 (DE3)	derived from BL21(DE3) [*E. coli* F^- *ompT hsdS*$_B$ (r_B^- m_B^-) *gal dcm* (DE3)]	[2]
S. marcescens		
CH-1	Wild type strain; clinical isolate	[3]
Δ*rssBA*	*rssBA* knockout mutant, Gm^r	This study
Δ*pvc*	*pvc* operon (Sma0021-0023) knockout mutant, Sm^r	This study
Δ*rssBA*-*pvc*	*rssBA* and *pvc* operon knockout mutant, Gm^r, Sm^r	This study
Plasmid		
pGEM-T Easy	TA cloning vector, Amp^r	Promega
pBluescript II SK(+/-)	Cloning vector, Amp^r	Stratagene
pUT-Sm	Suicide plasmid containing mini-Tn*5* (Sm^r); requires Pir protein for replication	[4]
pUT-Km1	Suicide plasmid containing mini-Tn*5* (Km^r); requires Pir protein for replication	[4]
pACYC184	Cloning vector, Tc^r, Cm^r	[5]
pEGFP-C3	GFPmut1 variant, CMV promoter, f1 origin, SV40 origin, pUC origin, kanamycin resistant gene	BD Bioscience Clontech
pBAD18RssA (H248A)	pBAD18 containing full length of $rssA^{H248A}$, arabinose regulation, Cm^r	[6]
pBAD24-Amp	pBAD24, arabinose regulation, Amp^r	[7]
pBAD33-Cm	pBAD33, arabinose regulation, Cm^r	[7]
pBAD24::Sm	pBAD24 with insertion of Sm^r cassette at *Hind*III site	[8]
pBAD24EGFP::Sm	pBAD24-*egfp*-Sm^r	[8]
pEGFP-RssBA::Sm	pBAD24-*egfp-rssB-rssA*-Sm^r	[8]
pEGFP-$RssB^{D51E}$-RssA::Sm	pBAD24-*egfp*-$rssB^{D51E}$-*rssA*-Sm^r	[8]
pEGFP-RssB-$RssA^{H248A}$::Sm	pBAD24-*egfp-rssB*-$rssA^{H248A}$-Sm^r	This study
pEGFP-$RssB^{D51E}$-$RssA^{H248A}$::Sm	pBAD24-*egfp*-$rssB^{D51E}$-$rssA^{H248A}$-Sm^r	This study
pEGFP-RssB-$RssA^{\Delta PPD}$::Sm	pBAD24-*egfp-rssB*-$rssA^{\Delta PPD}$-Sm^r	This study
pBAD24EGFP::Gm	pBAD24-*egfp*-Gm^r	[8]

Supplementary Table 1. Bacterial strains and plasmids used in this study (continued)

Strain or Plasmid	Genotype or relevant characteristics	Source or reference
Plasmid		
pEGFP-RssBA::Gm	pBAD24-*egfp-rssB-rssA*-Gm^r	This study
pEGFP-$RssB^{D51E}$-RssA::Gm	pBAD24-*egfp-rssB*^{D51E}-*rssA*-Gm^r	This study
pEGFP-RssB-$RssA^{H248A}$::Gm	pBAD24-*egfp-rssB-rssA*^{H248A}-Gm^r	This study
pEGFP-$RssB^{D51E}$-$RssA^{H248A}$::Gm	pBAD24-*egfp-rssB*^{D51E}-*rssA*^{H248A}-Gm^r	This study
pEGFP-RssB-$RssA^{\Delta PPD}$::Gm	pBAD24-*egfp-rssB-rssA*$^{\Delta PPD}$-Gm^r	This study
pRssBA	pACYC184-*rssB-rssA* with its own promoter	[9]
$pRssB^{D51E}$-RssA	pACYC184-*rssB*^{D51E}-*rssA*	This study
pRssB-$RssA^{H248A}$	pACYC184-*rssB-rssA*^{H248A}	This study
$pRssB^{D51E}$-$RssA^{H248A}$	pACYC184-*rssB*^{D51E}-*rssA*^{H248A}	This study
pRssB-$RssA^{\Delta PPD}$	pACYC184-*rssB-rssA*$^{\Delta PPD}$	This study
pRssB-$RssA^{chimeric}$	pACYC184-*rssB-qseC*$^{aa\ 1\text{-}189}$-*rssA*$^{aa\ 192\text{-}473}$	This study
pET28 series	Expression vector, His tag	Novagen
pPvc	pBAD33-*pvc* operon (Sma0021~0023)	This study
pBAD24-$RssA^{chimeric}$::Sm	pBAD24-*qseC*$^{aa\ 1\text{-}189}$-*rssA*$^{aa\ 192\text{-}473}$-Sm^r	This study
pBAD24-RssB-$RssA^{chimeric}$::Sm	pBAD24-*rssB-qseC*$^{aa\ 1\text{-}189}$-*rssA*$^{aa\ 192\text{-}473}$-Sm^r	This study
pEGFP-RssB-$RssA^{chimeric}$::Sm	pBAD24-*egfp-rssB-qseC*$^{aa\ 1\text{-}189}$-*rssA*$^{aa\ 192\text{-}473}$-Sm^r	This study
pET28b-RssA	pET28b-*rssA*	This study
pET28a-$RssA^{PPD}$	pET28a-*rssA*$^{aa\ 32\text{-}163}$	This study
pET28b-$RssA^{chimeric}$	pET28b-*qseC*$^{aa\ 1\text{-}189}$-*rssA*$^{aa\ 192\text{-}473}$	This study
pET28b-$RssA^{H248A}$	pET28b-*rssA*^{H248A}	This study
pET28cRssA	pET28c-*rssA*$^{aa\ 190\text{-}469}$	[6]
pBAD33-his28b-$RssA^{\Delta PPD}$	pBAD33-*rssA*$^{\Delta PPD}$ (his-tagged)	This study

Amp, ampicillin; Sm, streptomycin; Cm, chloramphenicol; Km, kanamycin; Gm, gentamicin; Tc, tetracycline

Supplementary Table 2. PCR primers used in this study

Primer	Sequence (5'–3')
PvcCF1	TAACAGGAGCTCATTGTGGACAGC
PvcCR2	GCTCTAGAGGGAAAGGATCACAGCGTGG
BamHRssAF1	CGGGATCCCATGATCGGTTTCAAATCCTTCTTTATGCG
HindRssAR2	CCCAAGCTTTGGTCGTGGTTCACTCTCAG
histagF	GCGGATAACAATTCCCCTCTAGA
SphIhistagR	GCATGCAGCCAACTCAGCTTCCTTTC
SalRssAhisF	GTCGACATGATCGGTTTCAAATCCTTCTTTATGCG
XhoRssAhisR	CTCGAGGGGTCGTGGTTCACTCTCAG
PstTM2F	CTGCAGACGATCGCACCACGCTGTTC
BamHPPDf1	GGATCCTATTGGTATTACCCCGACATGGAGAAA
SacPPDr1	GAGCTCATTGCCGAACAGCGTGGTGC
XbanQseCF1	GCTCTAGATGAAGCGTCTCAGCCTGCG
BamHQseCF1	CGGGATCCATGAAGCGTCTCAGCCTGCG
nQseCR1	CAGCTCGCGCCCGACCATC
cRssAF1	CGGCCGCTGCGCCAGATCG
PstcRssAR1	AAACTGCAGTCATGGTCGTGGTTCACTCTCAGTC
SmaRssBF1	TCCCCCGGGATGAACATATTGTTGGTGGAAGAC
XbaRssBR1	GCTCTAGACGCTACTCTTTCTTCAGCAAATAG
EcoEGFPF1	CGGAATTCATGGTGAGCAAGGGCGAGGAG
SmaEGFPR1	TCCCCCGGGGTACTTGTACAGCTCGTCCATGCC
FlhDCRTF	GCCAAAAGGAATGTTACCGT
FlhDCRTR	CAGTTGCGGCGAAAGTTTAC
16SrDNAF	AACTGGAGGAAGGTGGGGAT
16SrDNAR	AGGAGGTGATCCAACCGCA
RTsma0021F	TACGTGAATTATTGCAATACCGCC
RTsma0021R	GCAGATCATAATCATCGGTATGCT
RpoDRTF	AATCTCGATGGAAACGCCGA
RpoDRTR	ATACGCTCACGGGTAACGTC

Underlined sequences represent restriction endonuclease cutting sites.

Supplementary Methods

Construction of recombinant plasmid

To express the His-tagged N- and C-terminal of RssA, we amplified the fragment containing full-length *rssA* by using the primer pair BamHRssAF2/HindRssAR2. The PCR product was digested by BamHI and HindIII and cloned into the BamHI/HindIII sites of pET28b to form pET28b-RssA. To express the His-tagged, N-terminal periplasmic domain of RssA (RssA^{PPD}), a *rssA* fragment encoding amino acids 32–163 was amplified with the primer pair BamHPPDf1/SacPPDr1 and digested by BamHI and SacI. The digested fragment was further cloned into the BamHI/SacI sites of pET28a to form pET28a-RssA^{PPD}. pET28cRssA[6] was used to express the His-tagged cytoplasmic part of RssA (cRssA).

To express the His-tagged N- and C-temimal of RssA in *S. marcescens*, the fragment His28b, which comprised a coding region for hexa histidine-tags and multiple cloning sites from pET28b, was amplified with the primer pair histagF and SphIhistagR and cloned into pGEM-T-Easy to form pGEM-T-Easy-his28b. The fragment His28b was obtained by digestion of pGEM-T-Easy-his28b via BamHI and SphI, followed by cloning into the BamHI/SphI sites of pBAD33-Cm to form pBAD33-his28b. Full-length *rssA* without stop codon was amplified with the primer pair SalRssAhisF and XhoRssAhisR and digested using SalI and XhoI. The digested *rssA* fragment was cloned into the SalI-XhoI sites of pBAD33-his28b to form pBAD33-his28b-RssA. To construct the His-tagged N- and C-terminal of RssA$^{\Delta PPD}$ with deletion of amino acid (a.a.) 32 to 163 (predicted periplasmic domain), the TM2F-PPD fragment was amplified with the primer

pair PstTM2F and XhoRssAhisR and digested by PstI and XhoI. The digested TM2F-PPD fragment was cloned into the PstI-XhoI site of pBAD33-his28b-RssA to form pBAD33-his28b-RssA$^{\Delta PPD}$.

To express EGFP-tagged RssB (EGFP-RssB) and RssA in one plasmid, we used pEGFP-RssBA::Sm[8]. To investigate whether functional RssA or RssB influences RssAB signaling in response to different culture conditions, the unphosphorylated form of RssA (RssA^{H248A}), RssA deficient in the predicted periplasmic domain (a.a. 32 to 163) (RssA$^{\Delta PPD}$) and the unphosphorylated form of RssB (RssB^{D51E}) were used to replace RssA and RssB at pEGFP-RssBA::Sm. pEGFP-RssB-RssA^{H248A}::Sm and pEGFP-RssB^{D51E}-RssA^{H248A}::Sm were constructed by replacement of *rssA* in pEGFP-RssBA::Sm and pEGFP-RssB^{D51E}-RssA::Sm[8] with *rssA*^{H248A}, which was digested from pBAD18RssA (H248A)[6,8] using PstI and StuI, respectively. To construct RssA$^{\Delta PPD}$ with deleted amino acids 32 to 163, which represents the predicted periplasmic domain (PPD), the TM2F fragment was obtained by digestion of pBAD33-his28b-RssA via PstI and StuI and further ligated into the PstI-StuI sites of pEGFP-RssBA::Sm to form pEGFP-RssB-RssA$^{\Delta PPD}$::Sm. The Sm^{r} cassette in pBAD24-EGFP::Sm, pEGFP-RssB-RssA^{H248A}::Sm, pEGFP-RssB^{D51E}-RssA::Sm, pEGFP-RssB^{D51E}-RssA^{H248A}::Sm and pEGFP-RssB-RssA$^{\Delta PPD}$::Sm was replaced with the Gm^{r} cassette to form pBAD24-EGFP::Gm, pEGFP-RssB-RssA^{H248A}::Gm, pEGFP-RssB^{D51E}-RssA::Gm, pEGFP-RssB^{D51E}-RssA^{H248A}::Gm, and pEGFP-RssB-RssA$^{\Delta PPD}$::Gm, respectively.

The N-terminal periplasmic sensor domain of RssA (amino acids 1–192) was replaced with

189 amino acids of N-terminal QseC$^{\mathrm{aa\ 1\text{-}189}}$ to form the chimeric protein RssA^{chimeric}. The chimeric protein RssA^{chimeric} comprised the N-terminal periplasmic sensor domain of QseC[10] and the cytoplamic sensor kinase domain of RssA[6]. The *qseC*$^{aa\ 1\text{-}189}$ and *rssA*$^{aa\ 192\text{-}473}$ fragments, respectively encoding amino acids 1–189 of QseC and amino acids 192–473 of RssA from *S. marcescens* CH-1, were amplified with the primer pairs XbanQseCF1/nQseCR1 and cRssAF1/PstcRssAR1, respectively, followed by addition of phosphate groups via polynucleotide kinase (New England Biolabs, U.S.A.). The fragments of *qseC*$^{aa\ 1\text{-}189}$ and *rssA*$^{aa\ 192\text{-}473}$ were digested by XbaI and PstI, respectively. The digested fragments were further cloned into the XbaI/PstI sites of pBAD24::Sm to form pBAD24-RssA^{chimeric}::Sm. To express RssB and RssA^{chimeric} in one plasmid, a *rssB* fragment encoding full-length RssB was amplified with the primer pair SmaRssBF1/XbaRssBR1. The PCR product was digested by SmaI and XbaI, and cloned into the SmaI/XbaI sites of pBAD24-RssA ^{chimeric}::Sm to form pBAD24-RssB-RssA^{chimeric}::Sm. To express EGFP-RssB and RssA^{chimeric} in one plasmid, the *egfp* gene was amplified with the primer pair EcoEGFPF1 and SmaEGFPR1. The PCR product was digested by EcoRI and SmaI, and cloned into the EcoRI/SmaI sites of pBAD24-RssB-RssA^{chimeric}::Sm to form pEGFP-RssB-RssA^{chimeric}::Sm. To express the His-tagged N- and C-terminal of RssA^{chimeric}, fragment of *rssA*^{chimeric} was amplified with the primer pair BamHQseCF1 and HindRssAR2, digested by BamHI and HindIII, and subcloned the into BamHI/HindIII sites of pET28b to form pET28b-RssA^{chimeric}.

pRssBA[9], which contains full-length *rssBA* with its own promoter inserted in the SalI/XbaI

sites of pACYC184, was used to restore *rssBA*. pRssB^{D51E}-RssA, pRssB-RssA^{H248A}, pRssB^{D51E}-RssA^{H248A}, pRssB-RssA$^{\Delta PPD}$, and pRssB-RssA^{chimeric} were constructed by replacing wild-type *rssB* and/or *rssA* in pRssBA with *rssB*^{D51E}, *rssA*^{H248A}, *rssB*^{D51E}-*rssA*^{H248A}, *rssA*$^{\Delta PPD}$, and *qseC*$^{aa\ 1\text{-}189}$-*rssA*$^{aa\ 192\text{-}473}$. *S. marcescens* CH-1 and Δ*rssBA* harboring each recombinant plasmid were used to analyze swarming motility. To express the *pvc* cluster, the fragment containing the full-length *pvc* cluster was amplified with the primer pair PvcCF1 and PvcCR2, followed by digestion using SacI and XbaI. The digested fragment was further cloned into the SacI/XbaI sites of pBAD33-Cm to form pPvc.

Protein purification

To purify his-tagged form of full-length RssA, RssA^{H248A}, and RssA^{chimeric}, we diluted overnight culture of *E. coli* C41 (DE3)[2] carrying pET28b-RssA, pET28b-RssA^{H248A}, or pET28b-RssA^{chimeric} 1:100 into fresh LB broth containing 50 μg/ml kanamycin, and cultivated to early-log phase (OD_{600} around 0.3) at 37°C, followed by supplementation of 0.5 mM IPTG. For his-tagged RssA$^{\Delta PPD}$, overnight culture of *E. coli* C41 (DE3) carrying pBAD33-his28b-RssA$^{\Delta PPD}$ was diluted 1:100 into fresh LB broth containing 50 μg/ml kanamycin and cultivated to early-log phase (OD_{600} around 0.3) at 37°C, followed by supplementation of 0.2% arabinose. Bacteria were grown at 30°C for an additional 4 hrs, harvested in 8 ml of ice-cold PBS by centrifugation, and disrupted with a French press at 1200 psi for at least two passes at 4°C. Membrane vesicles were prepared and solubilized with 1% n-dodecyl-β-D-maltopyranoside (DDM) as previously described[11]. After removing the unsolublized material by ultracentrifugation at 100,000 × g at

4°C for 30 min, His-tagged RssA was purified from solubilized supernatant by Ni^{2+}-nitrilotriacetic acid (NTA) affinity chromatography according to the manufacturer's instructions (GE Healthcare Lifesciences, U.S.A.), prior to elution with ice-cold PBS containing 0.1% DDM and 250 mM imidazole. Purified His-tagged RssA was dialyzed against ice-cold reaction buffer (50 mM Tris-HCl, pH 8.0, 5 mM $MgCl_2$, 50 mM KCl, 0.2 mM dithiothreitol, or DTT, and 10% glycerol)[6]. To purify the RssA periplasmic sensor domain, overnight culture of *E. coli* BL21 (DE3) pLysS harboring pET28a-$RssA^{PPD}$ was diluted 1:100 into fresh LB broth containing 50 μg/ml kanamycin and 50 μg/ml chloramphenicol and cultivated at 37°C to early-log phase (OD_{600} around 0.3), followed by supplementation of 0.5 mM IPTG. Bacteria were grown at 30°C for an additional 4 hrs, harvested in 8 ml of ice-cold PBS by centrifugation, and disrupted with a French press at 10,000 psi for two passes at 4°C. The solubilized cytoplasmic part was collected by centrifugation twice at 13,000 × g at 4°C and applied to 0.45 μm filtration to remove unsolubilized material. His-tagged $RssA^{aa\ 32\text{-}163}$ was purified from clear supernatant by Ni^{2+}-NTA affinity chromatography and eluted with ice-cold PBS containing 0.1% DDM and 250 mM imidazole. The purified $RssA^{aa\ 32\text{-}163}$ was dialyzed against ice-cold 20 mM sodium phosphate (pH 7.4) and 0.2 mM DTT.

To purify protein from inclusion bodies, the unsolubilized inclusion bodies of French press-disrupted cells were isolated by centrifugation at 6,200 × g for 30 min at 4°C and washed once with a solution of 50 mM sodium phosphate (pH 8.0) and 300 mM sodium chloride. Inclusion bodies were resuspended in a solution of 20 mM sodium phosphate, 100 mM sodium chloride,

and 7 M urea, and incubated on ice for 1 hr. The unsolubilized material was removed by ultracentrifugation at 150,000 × g for 45 min. Supernatants were serially dialyzed against ice-cold sodium phosphate (pH 8.0) with decreasing concentration of urea (from 6 M to 0 M). The clear sample was filtered (0.45 μm) and applied to Ni^{2+}-NTA affinity column for purification of His-tagged RssA or $RssA^{aa\ 32\text{-}163}$. The purified RssA and $RssA^{aa\ 32\text{-}163}$ were dialyzed against ice-cold reaction buffer[6] and ice-cold 20 mM sodium phosphate (pH 7.4), 0.2 mM DTT, respectively. Purity of proteins was judged to be at least 95% based on a Coomassie blue staining assessment. Protein concentration was determined using the Bradford protein assay according to the manufacturer's instructions (Bio-Rad, U.S.A.).

Supplementary References

1 Herrero, M., de Lorenzo, V. & Timmis, K. N. Transposon vectors containing non-antibiotic resistance selection markers for cloning and stable chromosomal insertion of foreign genes in gram-negative bacteria. *J Bacteriol* **172**, 6557-6567 (1990).

2 Miroux, B. & Walker, J. E. Over-production of proteins in *Escherichia coli*: mutant hosts that allow synthesis of some membrane proteins and globular proteins at high levels. *Journal of molecular biology* **260**, 289-298 (1996).

3 Lai, H. C. *et al.* The RssAB two-component signal transduction system in *Serratia marcescens* regulates swarming motility and cell envelope architecture in response to exogenous saturated fatty acids. *J Bacteriol* **187**, 3407-3414 (2005).

4 de Lorenzo, V. & Timmis, K. N. Analysis and construction of stable phenotypes in gram-negative bacteria with Tn5- and Tn10-derived minitransposons. *Methods Enzymol* **235**, 386-405 (1994).

5 Chang, A. C. & Cohen, S. N. Construction and characterization of amplifiable multicopy DNA cloning vehicles derived from the P15A cryptic miniplasmid. *J Bacteriol* **134**, 1141-1156 (1978).

6 Wei, J. R. *et al.* Biochemical characterization of RssA-RssB, a two-component signal transduction system regulating swarming behavior in *Serratia marcescens*. *J Bacteriol* **187**, 5683-5690 (2005).

7 Guzman, L. M., Belin, D., Carson, M. J. & Beckwith, J. Tight regulation, modulation, and high-level expression by vectors containing the arabinose P_{BAD} promoter. *J Bacteriol* **177**, 4121-4130 (1995).

8 Tsai, Y. H. *et al.* RssAB signaling coordinates early development of surface multicellularity in *Serratia marcescens*. *PLoS One* **6**, e24154 (2011).

9 Lin, C. S. *et al.* RssAB-FlhDC-ShlBA as a major pathogenesis pathway in *Serratia marcescens*. *Infect Immun* **78**, 4870-4881 (2010).

10 Clarke, M. B., Hughes, D. T., Zhu, C., Boedeker, E. C. & Sperandio, V. The QseC sensor

kinase: a bacterial adrenergic receptor. *Proceedings of the National Academy of Sciences of the United States of America* **103**, 10420-10425 (2006).

11 Drew, D., Lerch, M., Kunji, E., Slotboom, D. J. & de Gier, J. W. Optimization of membrane protein overexpression and purification using GFP fusions. *Nat Methods* **3**, 303-313 (2006).
